# Integrating bulk RNA-seq and scRNA-seq analyses revealed the function and clinical value of thrombospondins in colon cancer

**DOI:** 10.1016/j.csbj.2024.05.021

**Published:** 2024-05-17

**Authors:** Jing Li, Ying Tang, Fei Long, Luyao Tian, Ao Tang, LiHui Ding, Juan Chen, Mingwei Liu

**Affiliations:** a Key Laboratory of Clinical Laboratory Diagnostics, College of Laboratory Medicine, Chongqing Medical University, Chongqing 400046, China; b Medical Laboratory, People's Hospital of Qingbaijiang District, Chengdu 61300, China; c Department of Laboratory Medicine, Zhongnan Hospital of Wuhan University, Wuhan, China; d Center for Single-Cell Omics and Tumor Liquid Biopsy, Zhongnan Hospital of Wuhan University, Wuhan, China

**Keywords:** Colon Cancer, Thrombospondins, Tumor microenvironment, *THBS2*, Cancer-associated fibroblasts

## Abstract

**Background:**

Acting as mediators in cell-matrix and cell-cell communication, matricellular proteins play a crucial role in cancer progression. Thrombospondins (TSPs), a type of matricellular glycoproteins, are key regulators in cancer biology with multifaceted roles. Although TSPs have been implicated in anti-tumor immunity and epithelial-mesenchymal transition (EMT) in several malignancies, their specific roles to colon cancer remain elusive. Addressing this knowledge gap is essential, as understanding the function of TSPs in colon cancer could identify new therapeutic targets and prognostic markers.

**Methods:**

Analyzing 1981 samples from 10 high-throughput datasets, including six bulk RNA-seq, three scRNA-seq, and one spatial transcriptome dataset, our study investigated the prognostic relevance, risk stratification value, immune heterogeneity, and cellular origin of TSPs, as well as their influence on cancer-associated fibroblasts (CAFs). Utilizing survival analysis, unsupervised clustering, and functional enrichment, along with multiple correlation analyses of the tumor-microenvironment (TME) via Gene Set Variation Analysis (GSVA), spatial localization, Monocle2, and CellPhoneDB, we provided insights into the clinical and cellular implications of TSPs.

**Results:**

First, we observed significant upregulation of *THBS2* and *COMP* in colon cancer, both of which displayed significant prognostic value. Additionally, we detected a significant positive correlation between TSPs and immune cells, as well as marker genes of EMT. Second, based on TSPs expression, patients were divided into two clusters with distinct prognoses: the high TSPs expression group (TSPs-H) was characterized by pronounced immune and stromal cell infiltration, and notably elevated T-cell exhaustion scores. Subsequently, we found that *THBS2* and *COMP* may be associated with the differentiation of CAFs into pan-iCAFs and pan-dCAFs, which are known for their heightened matrix remodeling activities. Moreover, *THBS2* enhanced CAFs communication with vascular endothelial cells and monocyte-macrophages. CAFs expressing *THBS2* (*THBS2*^+^ CAFs) demonstrated higher scores across multiple signaling pathways, including angiogenic, EMT, Hedgehog, Notch, Wnt, and TGF-β, when compared to *THBS2*^-^ CAFs. These observations suggest that *THBS2* may be associated with stronger pro-carcinogenic activity in CAFs.

**Conclusions:**

This study revealed the crucial role of TSPs and the significant correlation between THBS2 and CAFs interactions in colon cancer progression, providing valuable insights for targeting TSPs to mitigate cancer progression.

## Highlights

• Exploring thrombospondins' roles using bulk RNA-seq, scRNA-seq, spatial transcriptomics, cell line drug sensitivity, etc.
• We discovered that pan-dCAFs are major sources of TSPs in colon cancer. This CAF subtype has high matrix remodeling activity.
• Based on TSPs expression, patients can be divided into two clusters with distinct prognoses and immune microenvironment.
• We found *THBS2* may link to CAFs' pro-carcinogenic activity. THBS2^+^ CAFs showed stronger communication than THBS2^-^ CAFs.
• A web server presenting the main findings of this study is available at http://tsp.liumwei.org/Col.Cancer.

## Graphical Abstract

**Figure:**
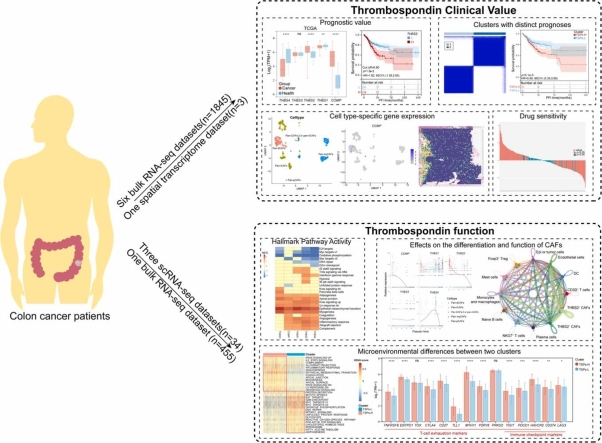

## 1 Introduction

Colon cancer, originating from the colon epithelium, is characterized by high heterogeneity and subtle early symptoms, leading to most diagnoses at advanced stages [1], [2]. Increasing evidence shows that molecular alterations in the extracellular matrix (ECM) remarkably impact immune response, angiogenesis, and epithelial mesenchymal transition within the tumor microenvironment [3], [4], [5], thereby influencing colon cancer progression. Cancer-associated fibroblasts, as one of the major sources of ECM, possess an extreme capacity for matrix remodeling [6], [7]. During this process, there is a notable increase in the expression of matricellular proteins [6]. These proteins, acting as a link between the ECM and cells, regulate cell-cell (including CAFs, monocyte-macrophages and tumor cells) and cell-matrix interactions [5], [6], [8], [9], impacting cellular functions and playing an important role in anti-tumor immunity and other processes. Given their overexpression and structural diversity, matricellular proteins are considered attractive targets for cancer therapy [5], [6], [8]. Notably, neutralising antibodies against matrix proteins such as CCN, tenascin, and SIBLINGs have shown promising results in preclinical cancer models, suggesting the feasibility of developing anti-matrix protein therapies against cancer [8].

Thrombospondins, a subgroup of matricellular proteins, are of particular interest due to their significant role in tumor progression [4], [5], [10]. This family can be divided into trimeric (*THBS1* and *THBS2*) and pentameric proteins (*THBS3*, *THBS4* and *COMP*) based on subunit composition and functional structural domains [11]. *THBS1* and *THBS2,* initially recognized for their natural anti-angiogenic properties through interactions with CD36 or CD47, with *THBS1* exhibiting a higher affinity [12], have been shown to increase under similar conditions [13], [14]. Recent studies have highlighted TSPs roles in anti-tumor immunity and EMT. For instance, in melanoma models, blocking the THBS1-CD47 interaction enhances the activity of natural killer (NK) cells [15] and M1 macrophages [16]. Similarly, the interaction itself suppresses the cytotoxic functionality of CD8^+^ T cells against tumor cells [17]. In lung cancer, the absence of *THBS1*, rather than *THBS2*, in dendritic cells increases the infiltration of both CD4^+^ T and CD8^+^ T lymphocytes, thereby enhancing anti-tumor immunity [18]. In gallbladder cancer, CAFs-secreted *THBS4* promotes cancer cell proliferation and EMT by activating Akt and phosphorylating HSF1 [19], [20]. However, the role of TSPs in colon cancer’s immune response and EMT has been less explored. Studies in colon cancer cell lines, such as CT26 and LoVo, have shown that overexpression of *THBS2* enhances cellular glycolysis and lactate production, facilitates M2 polarization of macrophages, blocks T-cell proliferation, and reduces T-cell cytotoxicity [21]. Furthermore, inhibiting *THBS2* expression significantly suppresses EMT and cancer cell metastasis [22]. In colon cancer tissues, high *COMP* expression correlates with a higher proportion of M0 and M2 macrophages and a lower proportion of CD8^+^ T cells compared to tissues with low *COMP* expression [23]. Further investigation into the impact of TSP family members on colon cancer's immune response and EMT is warranted.

Our study initially assessed the functional and stratification values of TSPs through function enrichment, drug sensitivity, and unsupervised clustering. Then, we further investigated the tumor microenvironment differences between clusters using pathway activity scores and immunoreactivity scores. Finally, we probed the possible mechanisms by which TSPs affect the colonic microenvironment utilizing functional enrichment, pathway activity scores, pseudo-time analysis and cellular communication. Our findings indicate that pan-dCAFs are a major source of TSPs in the colon cancer microenvironment, and *THBS2* may be linked to the CAFs differentiation and increased pro-cancer activity. These results provide a theoretical foundation for targeting the TSP family to postpone colon cancer progression.

## 2 Material and methods

**Fig. 1:**
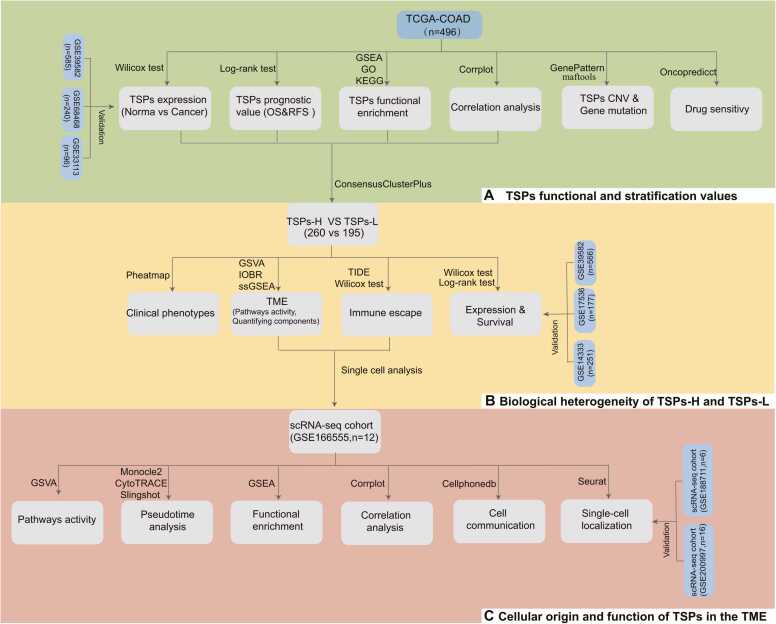
Workflow of study design. Our study consists of three main parts. First, we explored the role of TSPs in colon cancer through gene expression differences, unsupervised clustering, enrichment analysis and survival analysis. Next, we explored the effect of TSPs on the TME of colon cancer by pathway activity score, IOBR and TIDE analysis. Finally, we further explored how TSPs acts in the TME to impact colon cancer progression by pseudo-time analysis, cellular communication, GSEA and GSVA analysis.

The workflow of this study, illustrated in Fig. 1, includes three main Components: exploring the functional roles and stratification value of TSPs, investigating the differences in the tumor microenvironment between two clusters, and further dissecting the effects of TSPs on the TME using single-cell analysis. Below are concise descriptions of each methodological component, with more extensive details provided in Supplementary Material I.

### 2.1 Data acquisition and processing

The TCGAbiolinks was used to download colon cancer read count sequencing data and related clinical information from TCGA. The gene expression matrix of GSE39582, GSE68468, GSE14333, GSE17536, and GSE33113 were obtained from GEO. Using publicly available spatial transcriptome data, we identified the distribution of TSP family genes and CAFs marker genes in colon cancer[24].

### 2.2 Analyzing copy number, mutation, and protein expression

We visualized gene mutations with the oncoplot function of the maftools package, and examined the co-mutation and co-expression patterns between TSPs and other genes with the corrplot package. Additionally, we analyzed TSPs copy number variations with GenePattern. Using immunohistochemical images obtained from the Human Protein Atlas (HPA), we assessed the levels of TSP proteins in colon cancer tissues.

### 2.3 Enrichment and correlation analysis

Using Gene Set Enrichment Analysis (GSEA), we evaluated the biological functions affected by TSPs. To confirm the GSEA results, we identified 116 genes that are co-differentially expressed with five specific TSP molecules (Tables S1-S5) and performed GO and KEGG enrichment analyses. Additionally, we analyzed the correlations between TSPs and several factors including EMT, immune cells and molecules, matrix metalloproteinases and their inhibitors, as well as tissue-type and urokinase-type plasminogen activators.

### 2.4 Identifying molecular clusters based on TSPs expression

ConsensusClusterPlus was used for cluster identification, with datasets GSE14333, GSE17536, and GSE39582 serving as validation sets.

### 2.5 Comparing differences in tumor microenvironment between two clusters

We used GSVA to quantify the activity of Hallmark pathways. The single sample Gene Set Enrichment Analysis (ssGSEA) algorithm was used to quantify the abundance of immune cells. To confirm differences in immune cell composition between two clusters, we utilized the Immuno-Oncology Biological Research (IOBR) package[25], which integrates eight commonly used methods for estimating immune status. Furthermore, we performed Tumor Immune Dysfunction and Exclusion (TIDE) analysis to evaluate the likelihood of immune escape.

### 2.6 Single cell RNA-seq analysis

scRNA-seq datasets were processed with Seurat, and individual cell clusters were defined based on cell-specific marker genes from the literature or the CellMarker database (**FigS7A**)[26], [27], [28], [29]. Moreover, we utilized the Monocle2 algorithm to analyze the pseudo-time trajectory of CAFs and applied the corrplot package to examine the correlation between TSPs and point one branch genes.

### 2.7 Analyzing cell communication strength and pathway activity scores

We initially examined the effect of *THBS2* on the communication between CAFs and other cells using CellPhoneDB [30]. We divided CAFs into two groups: *THBS2*^+^ CAFs and *THBS2*^-^ CAFs, with the former expressing *THBS2* and the latter not. Additionally, we compared the gene expression of T cell exhaustion markers [31], [32] between the THBS2-H and THBS2-L groups to demonstrate the potential immunosuppressive effect of *THBS2*. Finally, we compared the Hallmark pathway activity score between *THBS2*^+^ and *THBS2*^-^ CAFs using GSVA.

### 2.8 Drug sensitivity analyses

We predicted patient response to 198 drugs from Genomics of Drug Sensitivity in Cancer 2 (GDSC2) using the R package oncoPredict.

### 2.9 Statistical analyses

Statistical analysis was performed using R software version 4.0.2. Between-group differences and survival analysis were partly conducted using SangerBox. The tools and sources used are listed in Table S6.

### 2.10 Webserver development

A dedicated webserver presents the study's findings, accessible at http://tsp.liumwei.org/Col.Cancer.

## 3 Results

### 3.1 Dysregulated expression and prognostic value of TSPs in colon cancer

**Fig. 2:**
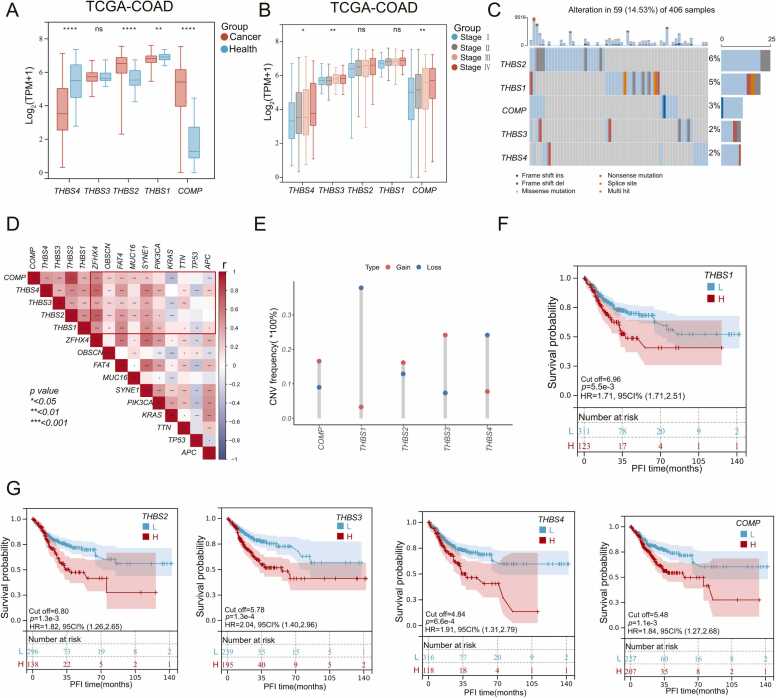
Heterogeneity of TSPs expression and prognostic value in colon cancer. **(A)** Differential expression of TSPs in colon cancer and normal tissues. ns, no significance, * *p* < 0.05, * * *p* < 0.01, * ** *p* < 0.001, * ** * *p* < 0.0001. **(B)** Differential expression of TSPs in different tumor stages. * *p* < 0.05, * * *p* < 0.01. **(C)** Mutations in TSPs within the TCGA-COAD cohort, analyzed by maftools. **(D)** The co-expression of TSPs with the top 10 mutated genes in the TCGA-COAD cohort, with box colors reflecting correlation coefficients. **(E)** TSPs Copy number variation in the TCGA cohort. **(F&G)** Differences in RFS between patients in the high and low expression groups of *THBS1*, *THBS2*, *THBS3*, *THBS4* and *COMP* in the TCGA cohort.

We initially examined TSPs expression in colon cancer. Except for *THBS3*, the expression of other TSPs was significantly different between health and cancer tissue (Fig. 2**A**). Among these, *THBS2* and *COMP* were upregulated in cancer tissue. Subsequently, we assessed the expression differences of TSPs at different tumor stages. All TSPs gradually increased in expression from stage I to IV, and *THBS3*, *THBS4* and *COMP* were statistically significant (Fig. 2**B &** Table S7). To validate the expression difference between health and cancer tissue, we analyzed TSPs expression in three additional independent cohorts (Fig.S1A). Obviously, TSPs expression was not completely consistent among different cohorts, but *THBS2* and *COMP* were significantly up-regulated across all four independent cohorts. These findings suggest a strong association between these two molecules and cancer progression, consistent with previous findings[33], [34], [35].

Gene mutations can alter gene expression by affecting protein coding sequences, gene readability, RNA splicing, and stability. Therefore, we further explored TSPs mutations. It can be seen that the mutation rate of the five TSP family molecules was relatively low (Fig. 2**C**). Additionally, we examined the co-occurrence of mutations in TSPs and colon cancer mutation-prone genes (**Fig.S1B**), and found that TSPs coexisted mutationally with *MUC16*, *FAT4*, *OBSCN*, and *ZFHX4* (**Fig.S1C**). In addition, most of these mutation-prone genes were also positively associated with TSPs expression (Fig. 2**D**). It has been reported that *MYC* and *TP53* significantly affect *THBS1* expression [36]. Hence, we further explored the effects of proto-oncogenes and tumor suppressor genes on TSPs expression. The correlation heatmap showed that *MYC* and *KRAS* were significantly correlated with the expression of TSPs (**Fig.S1D**). We then examined TSPs copy number variation. Clearly, *THBS1* and *THBS4* are more susceptible to copy number loss, while other molecules are more prone to copy number gain (Fig. 2**E**).

In addition, we explored differences in the protein levels of TSPs in healthy and cancer tissue. Our findings revealed that cancer had lower expression of THBS1, THBS3, and THBS4 compared to normal tissue, whereas the expression of THBS2 was higher (**Fig.S2A-D**). These changes were consistent with the alterations in mRNA levels of *THBS1* and *THBS2*. We then explored the prognostic value of individual TSPs. The higher expression of TSPs is associated with worse overall survival and recurrence-free survival (Fig. 2**F-G &** Fig.S1**E**). Therefore, TSPs was a risk factor for overall survival and recurrence-free survival in colon cancer.

### 3.2 Significant positive association between TSPs and marker genes related to epithelial-mesenchymal transition and immune cell infiltration

**Fig. 3:**
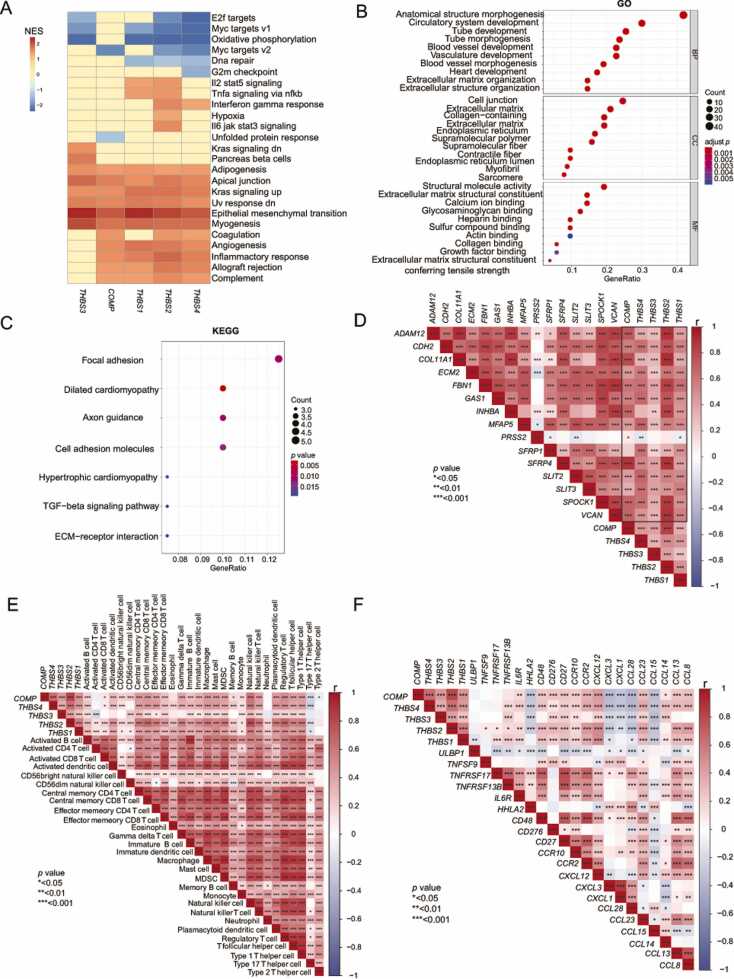
Significant positive association between TSPs and marker genes related to epithelial-mesenchymal transition and immune cell infiltration. **(A)** The heatmap displayed the biological pathways significantly enriched by the five TSPs molecules. The pathways exhibited in the heat map all met *|NES|* > 1 and *p.adjust* < 0.05. (B*&*C) The dotplot displayed the biological pathways significantly enriched by TSPs. (D) Co-expression of TSPs with differentially expressed EMT marker genes in colon cancer. (E&F) Correlation analysis of TSPs with immune cells and the top 20 molecules identified by ssGSEA, sorted by *FDR*.

As TSPs had a significant association with patient prognosis，we next explored the functional pathways related to TSPs. As shown in the heatmap, TSPs were significantly related to patients' immune function and EMT, as well as other processes that were involved in cancer progression (Fig. 3**A &** Table S8). To confirm the functional pathways associated with TSPs, we conducted GO and KEGG enrichment analysis. Using the intersection of each TSPs differential genes (Fig.S3A) and then performing functional enrichment, the results were consistent with GSEA (Fig. 3**B-C**). Additionally, we obtained 20 protein molecules closely associated with TSPs function from the STRING database (Fig.S3B). Likewise, the function of these molecules was highly correlated with the invasion and progression of colon cancer. In detail, FN1, TIMP1, TIMP2, MMP2, ITGA5, ITGAV, SDC1, and PLAT are related to metastasis and invasion; CD36 and CD47 are related to angiogenesis; CD47 is related to immune function; and LRP5 and LRP1 are related to lipid metabolism. The above results indicate that TSPs may play a crucial role in colon cancer invasion and progression.

To further characterize the effect of TSPs on EMT, we explored the correlation between TSPs and EMT marker genes. The results revealed a positive and statistically significant correlation between TSPs and these marker genes, with *THBS2* and *COMP* showing the strongest correlation (Fig. 3**D**). As is well known, matrix metalloproteinases (*MMP*), matrix metalloproteinase inhibitors (*TIMP*), tissue-type plasminogen activator (*TPA*), and urokinase-type plasminogen activator (*UPA*) play a crucial role in cancer invasion and progression [37]. There has been evidence that *THBS1* can increase the activity of *MMP* by inhibiting the production of *TIMP1* and enhancing the expression of *MMP2*, *MMP7*, and *MMP9* in T cells through the action of integrins [38]. To determine whether TSPs influence colon cancer progression, we explored the co-expression between TSPs and *MMP*, *TIMP*, *TPA* and *UPA*. The results showed that TSPs was highly correlated with *TIMP*, *MMP*, *TPA* and *UPA* (especially *THBS2* and *COMP*), and the correlation was essentially positive (Fig.S3C). In summary, it is possible that TSPs are associated with colon cancer invasion and progression.

The results above indicate that TSPs were linked to the activity of immune-related pathways. Therefore, we further explored the correlation between TSPs and immune cells. The heatmap showed a positive correlation between TSPs and most immune cells (Fig. 3**E**). Interestingly, we found that *THBS1* and *THBS2* were strongly associated with immune cells. Following that, we examined the relationship between TSPs and immune molecules. As shown, TSPs exhibited a positive correlation with most immune molecules (Fig. 3F & Fig.S4A). Once again, *THBS2* showed the most significant correlation with immune molecules.

### 3.3 Significant difference in relapse-free survival between patients in TSPs-H and TSPs-L groups

**Fig. 4:**
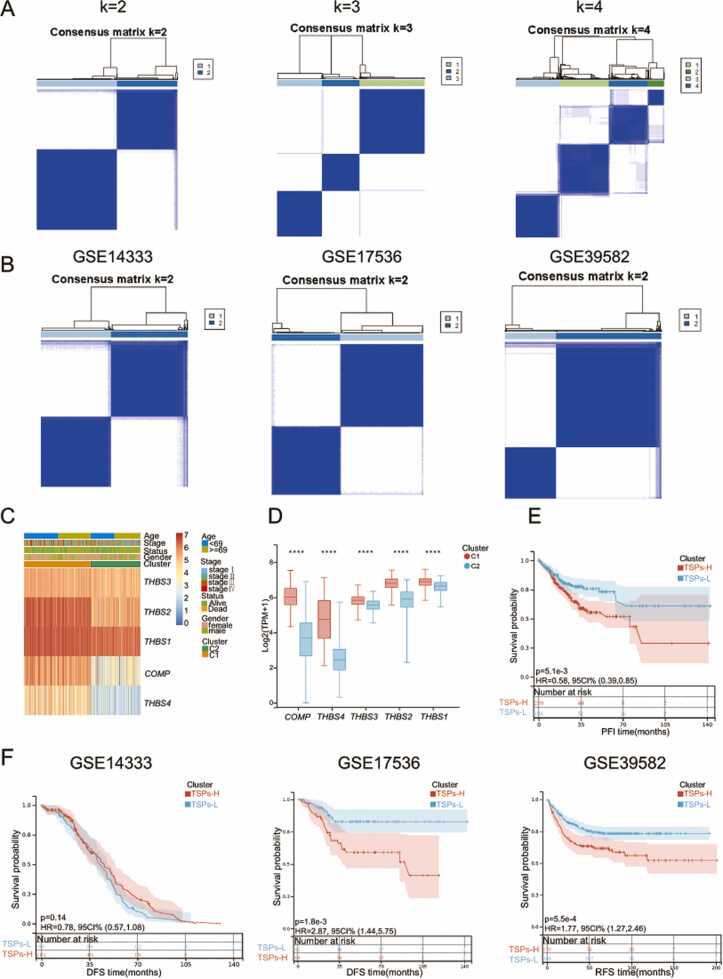
Based on TSPs expression, patients can be divided into two clusters with different prognoses. **(A)** Stratification of colon cancer patients in the TCGA cohort based on TSPs expression at different K values. **(B)** Multiple cohorts have demonstrated the effective classification of patients into two clusters based on TSPs expression. **(C)** Clinical phenotypic differences between the two clusters of patients in the TCGA cohort. **(D)** Differential expression of five TSPs molecules in two clusters of patients in the TCGA cohort. Significance was determined by Wilcoxon rank-sum test, * ** * *p* < 0.0001. **(E)** Differences in RFS between the two clusters of patients in the TCGA cohort. The TSPs-H group had higher expression of five TSPs molecules. **(F)** Differences in relapse-free survival between the two clusters of patients in the GSE14333, GSE17536 and GSE39582.

The results above suggest that TSPs may be linked to colon cancer progression. We then explored the stratification value of the TSP family. By unsupervised clustering, patients were divided into different clusters based on TSPs expression. In order to demonstrate the rationality of stratifying patients based on TSPs expression, we first applied ConsensusClusterPlus for unsupervised clustering in three additional colon cancer cohorts. As we can see, patients were well divided into two clusters (Fig. 4A-B & Fig.S5A). In addition, we compared the clinical characteristics of two clusters. The heatmap revealed an asymmetric distribution concerning tumor stage and survival status (Fig. 4**C**). Boxplot showed that the expression of five TSPs molecules in subtype C1 were significantly higher than that in subtype C2 (Fig. 4**D**). Thus, we designated the C1 subtype as TSPs-H group and the C2 subtype as TSPs-L group. Significant expression differences between the two clusters were also observed in the GSE14333, GSE17536, and GSE39582 cohorts (**Fig.S5B**). Lastly, we compared the recurrence-free survival of patients between TSPs-H and TSPs-L in TCGA and three GEO cohorts. The Kaplan-Meier curves showed that there was a significant difference in recurrence-free survival between two clusters, with TSPs-H group correlating with a shorter relapse-free survival (RFS) (Fig. 4**E-F**).

### 3.4 Significant differences in the tumor immune microenvironment between TSPs-H and TSPs-L patients

**Fig. 5:**
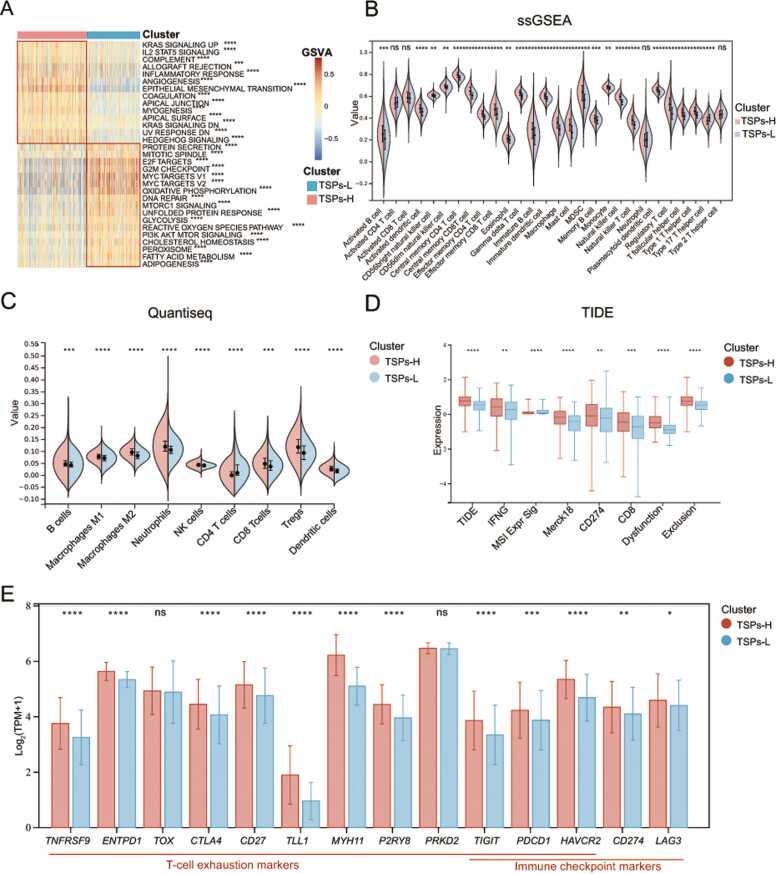
Significant differences in the tumor immune microenvironment between TSPs-H and TSPs-L patients. **(A)** GSVA analysis was utilized to assess Hallmark pathways score differences between the two clusters. * ** *p* < 0.001, * ** * *p* < 0.0001. **(B&C)** Differences in infiltrating cell component between two clusters were calculated by ssGSEA and quantiseq. **(D)** Calculation of immune escape by TIDE for two clusters. Merck18 is a T-cell inflamed signature from literature (Ayers M,2017). **(E)** Expression levels of T cell exhaustion and immune checkpoint-related genes among clusters. Bar heights represent the mean values, error lines illustrate the range of data fluctuations. * *p* < 0.05, * * *p* < 0.01, * ** *p* < 0.001, * ** * *p* < 0.0001.

To explore the reasons for the difference in relapse-free survival between two clusters, we applied GSVA to assess the activity of the hallmark pathways. We observed that the pathway activity scores between the two clusters were significantly different (Table S9). According to the heatmap, the score of immune-related pathways was significantly higher in the TSPs-H group. Additionally, pathways related to matrix activation and migration progression also exhibited significantly higher scores (Fig. 5**A**).

Given the contradiction between high immune pathway activity scores and poor prognosis in the TSPs-H group, we investigated tumor microenvironment differences between these two clusters. Initially, we assessed the differences in infiltrating cells between the two clusters of patients using ssGSEA (Table S10). The results showed that the infiltration scores of various immune cells in the TSPs-H group were significantly higher than those in the TSPs-L group, but the scores of immunosuppressive cells such as regulatory T cells and MDSC were also higher. (Fig. 5**B**). To mitigate algorithmic biases, we performed several algorithms (quantiseq, Timer, Ips, Mcp, EPIC, and xcell) to quantify various cell infiltrations (Fig. 5C & Fig.S6A-B, Table S11). Violin plots indicated that the TSPs-H group indeed had significantly elevated scores for immune cells. However, scores for CAFs, endothelial cells, and other stromal cells were also markedly higher. Furthermore, the xCell score revealed that the TSPs-H group exhibited increased scores for immune components, matrix, and the tumor microenvironment. Thus, TSPs may be associated with immune cell infiltration. The significant positive correlation of TSPs with immune molecules and immune cells and the upregulation of matrix activity and immune-related pathways activity by GSEA also support the above view (Fig. 3**A &** Fig. 3**E-F**).

Studies have demonstrated that the immunosuppressive effect of the stroma promotes tumor progression and immunotherapy resistance [39], [40], [41]. For example, CAFs suppress immune response by directly inhibiting the activation of CD8^+^ T cells and NK cells via upregulating the expression of immune checkpoint molecules (such as PD-1, PD-L1 and PD-L2) [40], [41]. As a result, we assume that the stroma Components in the TME of colon cancer within the TSPs-H group inhibit the function of immune cells, which adversely affects patients’ prognosis for recurrence-free survival. To verify our hypothesis, we first compared the TIDE scores of two clusters to evaluate whether immune escape exists (Table S12). As shown in the box plot, although the scores of IFNG and Merck18 were higher in the TSPs-H group, its TIDE score, Dysfunction score, Exclusion score, and *CD274* molecular level were also higher (Fig. 5**D**). Accordingly, patients in the TSPs-H group may exhibit greater levels of immune dysfunction and enhanced immune evasion. We further evaluated the expression of T cell exhaustion-related genes and immune checkpoint-related genes in two clusters (Table S13). Evidently, the expression levels of these marker genes were significantly upregulated in the TSPs-H group (Fig. 5**E**), suggesting a more severe case of T cell exhaustion and immune escape.

### 3.5 Fibroblasts are the main source of the TSP family in the colon cancer

**Fig. 6:**
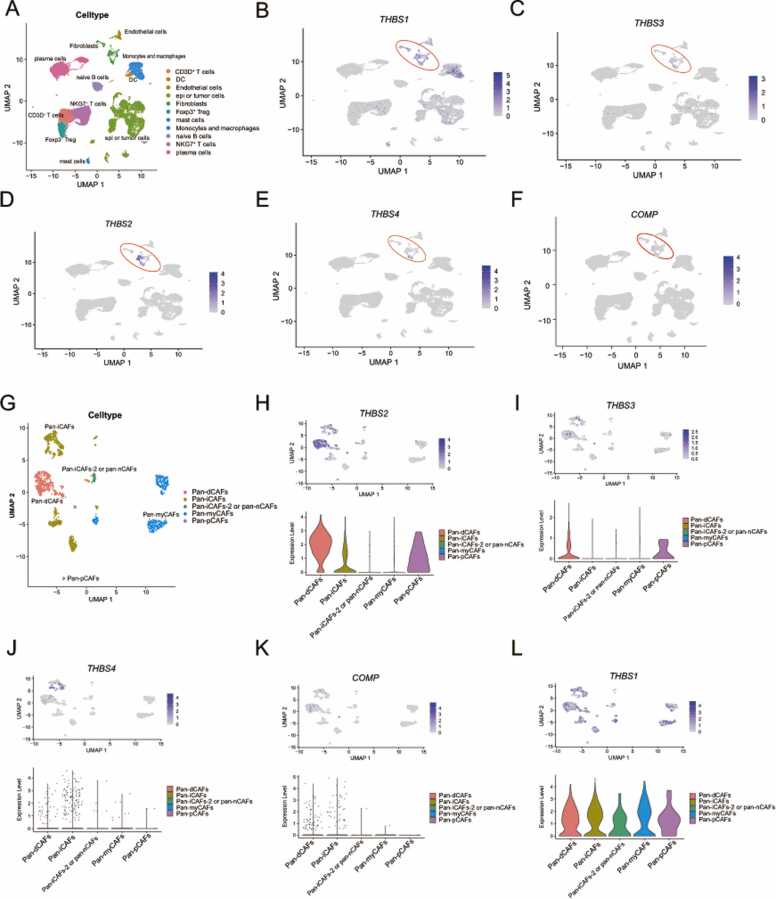
Single-cell analysis to localize the cellular origin of TSPs molecules in colon cancer. **(A)** Major cell types in the colon cancer microenvironment of the GSE166555 cohort. **(B&F)** Differential expression of five TSPs molecules across various cell types in dataset GSE16655. Each dot represents a cell. **(G)** Major CAFs subtypes in the colon cancer microenvironment of the GSE166555 cohort. **(H&L)** UMAP and violin plots clearly demonstrate differential expression of TSPs across different CAFs subtypes in the GSE16655 dataset. Each dot represents a cell.

To further investigate how TSPs regulates stromal and immune activity and affects patient survival, we used single cell analysis to localize the cellular origin of TSPs. To start, we examined TSPs expression in dataset GSE166555. Using cell-specific marker genes (Fig.S7A), we identified 11 different cell types (Fig. 6**A**). As depicted in UMAP plots, *THBS2*, *THBS4* and *COMP* were primarily expressed by fibroblasts, while *THBS3* was predominantly expressed by tumor cells and fibroblasts, and *THBS1* was predominantly expressed by fibroblasts, monocyte-macrophages and endothelial cells (Fig. 6**B-F**).

We then analyzed two other single-cell cohorts to minimize potential biases related to the sample collection site and the experimental procedure. The violin plot clearly indicated that the results of three scRNA-seq cohorts were generally consistent (Fig.S7B-C). Generally, *THBS1* was primarily found in fibroblasts, endothelial cells, and mononuclear macrophage cells, *THBS3* was mostly expressed in fibroblasts, tumor cells and T cells, whereas *THBS2*, *THBS4* and *COMP* were mainly found in fibroblasts. Furthermore, the spatial co-localization of CAFs marker genes with *THBS2*, *THBS4*, and *COMP* in colon cancer supports these findings (Fig.S8A).

### 3.6 *THBS2* may be associated with CAFs phenotype conversion

The fibroblasts in the TME play a crucial role in the progression of colon cancer. Given that different fibroblast subtypes have opposite effects on cancer progression [42], we continued our investigation into the expression of TSPs in distinct CAF subtypes. We extracted fibroblasts from the GSE166555 cohort and defined them based on CAFs markers commonly found in the literature [43], [44]. As shown in dotplot, all fibroblast subtypes were positive for CAFs markers (*RUNX1*, *COL1A2*, *VCAN*, *FAP*, *TAGLN* and *ACTA2*), indicating that fibroblasts in the TME were CAFs (**Fig.S7D**). There were five CAFs subtypes in this cohort (Fig. 6**G**). As we can see, *THBS1* was uniformly expressed in each subtype, *THBS2* was primarily expressed in pan-dCAFs, pan-iCAFs and pan-pCAFs. *THBS3* was primarily expressed in pan-dCAFs and pan-pCAFs. *THBS4* and *COMP* were mainly expressed in pan-dCAFs and pan-iCAFs (Fig. 6**H-L**).

**Fig. 7:**
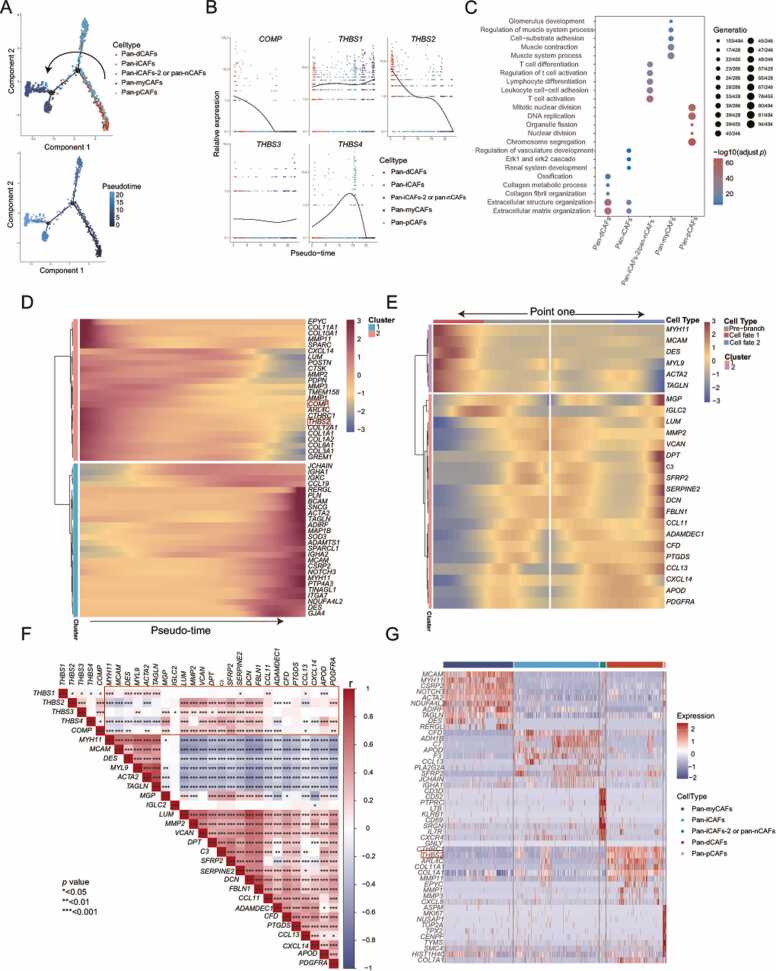
***THBS2*** may be associated with CAFs phenotype conversion in the colon cancer microenvironment. **(A)** The cell trajectory of CAFs generated by monocle2 is colored by each subtype (top) and pseudotime (bottom). **(B)** Changes in the expression of the five TSPs molecules within each CAFs subtypes as pseudo-time proceeds. Each dot represents an individual cell. **(C)** Gene Ontology enrichment analysis of five CAFs subtypes. **(D)**Heatmap showing the top 50 genes with the largest changes in expression as pseudo-time proceeds. Arrows indicate the direction of pseudo-time progression. The genes exhibited in the heat map all met *q* < 0.01. **(E)** Heatmap showing changes in the expression of key genes in the point one branch node over pseudo-time. The genes exhibited in the heat map all met *q* < 0.01. **(F)** Co-expression of TSPs with point one branch genes. * *p* < 0.05, * * *p* < 0.01, * ** *p* < 0.001**. (G)** Heatma*p* showing the top 10 DEGs among five CAFs subtypes in the GSE166555 cohort. The genes exhibited in the heat map all met log_2_FC > 1.8 and *p* < 0.01.

To determine the distribution of TSPs in each CAFs subtypes, we analyzed another single-cell cohort, defining each population based on the same CAFs markers. The distribution of TSPs in each CAFs subtypes of this cohort was basically consistent with GSE166555 (Fig.S7E). Combining the results of two independent cohorts, we can draw the following conclusions: *THBS1* was consistently expressed in every CAFs subtypes, whereas other TSPs were mainly expressed in pan-dCAFs. Subsequently, to investigate the effect of differential TSPs expression in each subtype on the phenotype conversion of CAFs, we conducted pseudo-time analysis. Based on the results of monocle2, cytoTRACE and Slingshot, we believed that the potential differentiation direction was from pan-iCAFs and pan-dCAFs to pan-myCAFs (Fig. 7A & Fig.S8B-C). Notably, *THBS2* and *COMP* exhibited significant downregulation along the differentiation direction (Fig. 7**B**). This indicates that the two molecules might be associated with CAFs differentiation. On the basis of the enrichment analysis, we explored the function of CAFs subtypes and found that pan-dCAFs and pan-iCAFs were significantly associated with functional pathways linked to cancer progression, such as ECM remodelling, angiogenesis and ERK pathways (Fig. 7**C &** Table S14). This finding was generally consistent with the previous study [43].

We further assessed the impact of differential expression of TSPs on CAFs differentiation. Firstly, we screened the top 50 genes with significant impact on the cell trajectory and found that the expression levels of *THBS2*, *COMP* and markers associated with cancer progression (e.g. *POSTN*, *COL11A1*, *MMP11*, *MMP2*, *CTHRC1*, etc.) progressively decreased as the pseudo-time advanced (namely pan-dCAFs and pan-iCAFs to pan-myCAFs) (Fig. 7**D &** Table S15). Secondly, we investigated the co-expression of TSPs and point one branch-related genes. Cell fate one corresponds to state two, and cell fate two corresponds to state five (Fig.S8D). compared to cell fate one, we can clearly see that cluster one genes expression increased significantly in cell fate two, while cluster two genes expression decreased remarkably (Fig. 7**E**). The results above found that the expression of *THBS2* and *COMP* gradually reduced over time (Fig. 7**D**). Correlation analysis showed that these two genes have strong ties to genes in point one branch. Additionally, they showed a significant positive correlation with genes in cluster one and a pronounced negative correlation with genes in cluster two (Fig. 7**F**). Therefore, we speculate that the high expression of *THBS2* and *COMP* in colon cancer may be associated with CAFs phenotype conversion. Interestingly, we previously identified that the main sources of *THBS2* and *COMP* were pan-dCAFs and pan-iCAFs. We also discovered the genes that were significantly differentially expressed in several CAFs subtypes. Once again, we found that pan-dCAFs expressed significantly higher levels of *THBS2* than other clusters. In addition, pan-dCAFs expressed high levels of *COL11A1*, *COL1A1*, *MMP11* and *MMP1*, which are closely related to cancer invasion[45] (Fig. 7**G**). The above results—encompassing expression levels, functional enrichment, and pseudo-time analyses—consistently highlight THBS2's prominent role in colon cancer progression.

### 3.7 *THBS2* may be associated with enhanced tumor-promoting activity of CAFs

**Fig. 8:**
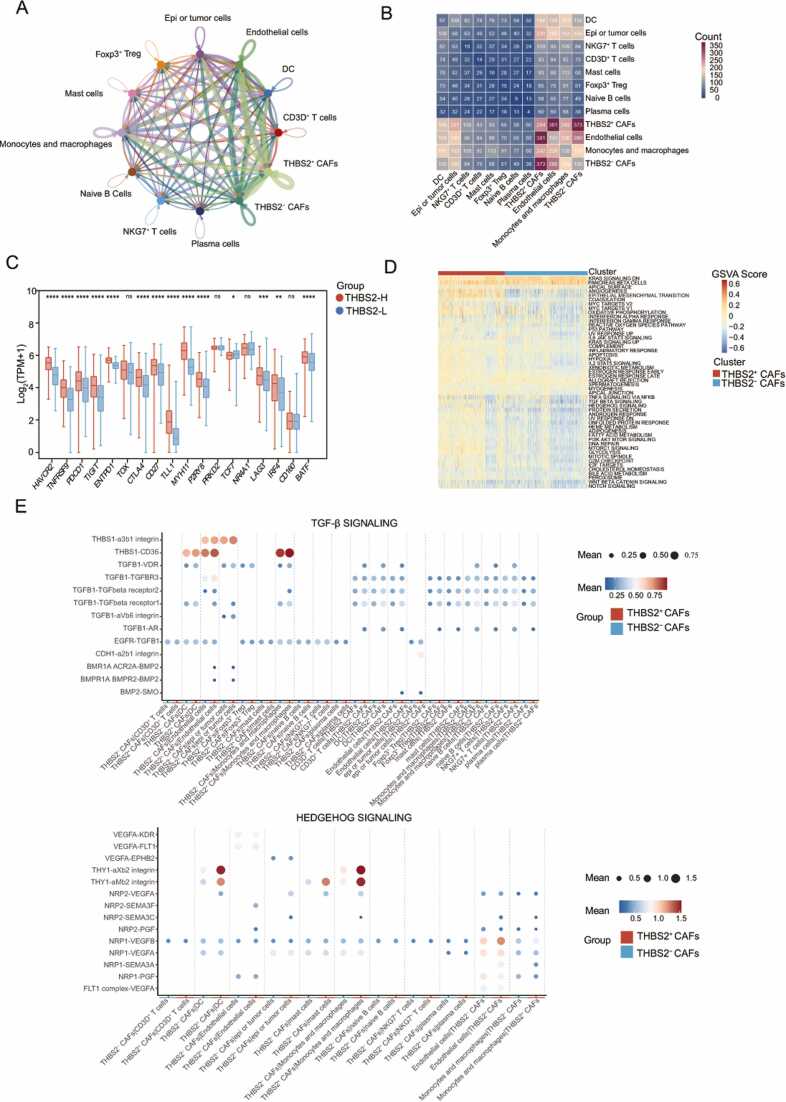
Effect of *THBS2* on the function of CAFs in the TME. **(A)** The impact of *THBS2* on the communication of CAFs with other cells in the tumor microenvironment (GSE166555). Significant cell communication was calculated based on the normalized cell matrix achieved by Seurat (*p* < 0.05). (**B**) The number of receptor-ligand pairs between various cell types was displayed by heatmap. **(C)** Box plot showing the expression of T-cell exhaustion markers in THBS2-H and THBS2-L groups within the TCGA cohort. **(D)** Differences in Hallmark pathway score between *THBS2*^+^ and *THBS2*^-^ CAFs assessed through GSVA within the TCGA cohort. **(E)** Differences in the mean expression levels of receptor-ligand pairs involved in the Hedgehog and TGF-β signaling pathways between *THBS2*^+^ CAFs and *THBS2*^-^ CAFs in dataset GSE16655. *THBS2*^+^ CAFs expressed *THBS2*, whereas *THBS2*^-^ CAFs did not.

In light of the above, *THBS2* may be associated with the differentiation of CAFs, and we further explored its role in CAFs function. Firstly, we employed CellPhoneDB to assess whether *THBS2* enhances communication between CAFs and other cells within the tumor microenvironment. We observed that the communication intensity between *THBS2*^+^ CAFs and other cells was stronger than that in *THBS2*^-^ CAFs, and the corresponding receptor-ligand heatmap appeared more pronounced (Fig. 8**A-B**). Moreover, we found that CAFs communicated most frequently with vascular endothelial cells, monocyte-macrophages, and tumor cells.

Studies have shown that CAFs affect the function and behavior of stromal cells in the TME by secreting growth factors, cytokines and matrix-degrading enzymes, thereby promoting tumor progression [46].In our study, we observed that CAFs have the most frequent communication with vascular endothelial cells, monocytes and macrophages, as well as tumor cells. Single-cell analysis identified pan-dCAFs with high matrix remodelling activity as the main source of *THBS2*. Furthermore, *THBS2* showed significant positive correlations with most markers of immune cells and immune molecules (Fig. 3E-F & Fig.S4). Based on these findings, we speculate that *THBS2* might be helpful for an immunosuppressive microenvironment. To validate this hypothesis, we compared the functional state of T cells in patients between THBS2-H and THBS2-L groups. The results showed that most T-cell exhaustion marker genes were significantly higher in THBS2-H group (Fig. 8**C**). Subsequently, GESA was utilized to investigate the biological processes associated with *THBS2*. We found that *THBS2*^+^ CAFs were significantly positively enriched in several pro-cancer pathways including angiogenesis, EMT and glycolysis (Fig.S9A).

After that, we investigated the effects of *THBS2* on the activity of common pathways. First, we compared the scores of Hallmark pathways by GSVA between two groups (Table S16). As shown in the heat map, most of the Hallmark pathway scores were significantly higher in *THBS2*^+^ CAFs compared to *THBS2*^-^ CAFs (Fig. 8D & Fig.S9B). Compared with *THBS2*^-^ CAFs, *THBS2*^+^ CAFs exhibited higher scores in metabolic pathways, classical tumor-promoting pathways, hypoxia, immune response, and apoptosis. Then, we compared the intensity of several common cancer signaling pathways in the *THBS2*^+^ CAFs group with that in the *THBS2*^-^ CAFs group (Table S17-S18). We found that the mean expression levels of receptor-ligand pairs involved in the Hedgehog signaling pathway, Notch signaling pathway, TGF-β signaling pathway and Wnt/β-Catenin Signaling pathway were higher in the *THBS2*^+^ CAFs (Fig. 8E & Fig.S10). These results were consistent with the previous GSVA findings.

### 3.8 *THBS2* may be associated with drug response in colon cancer patients

**Fig. 9:**
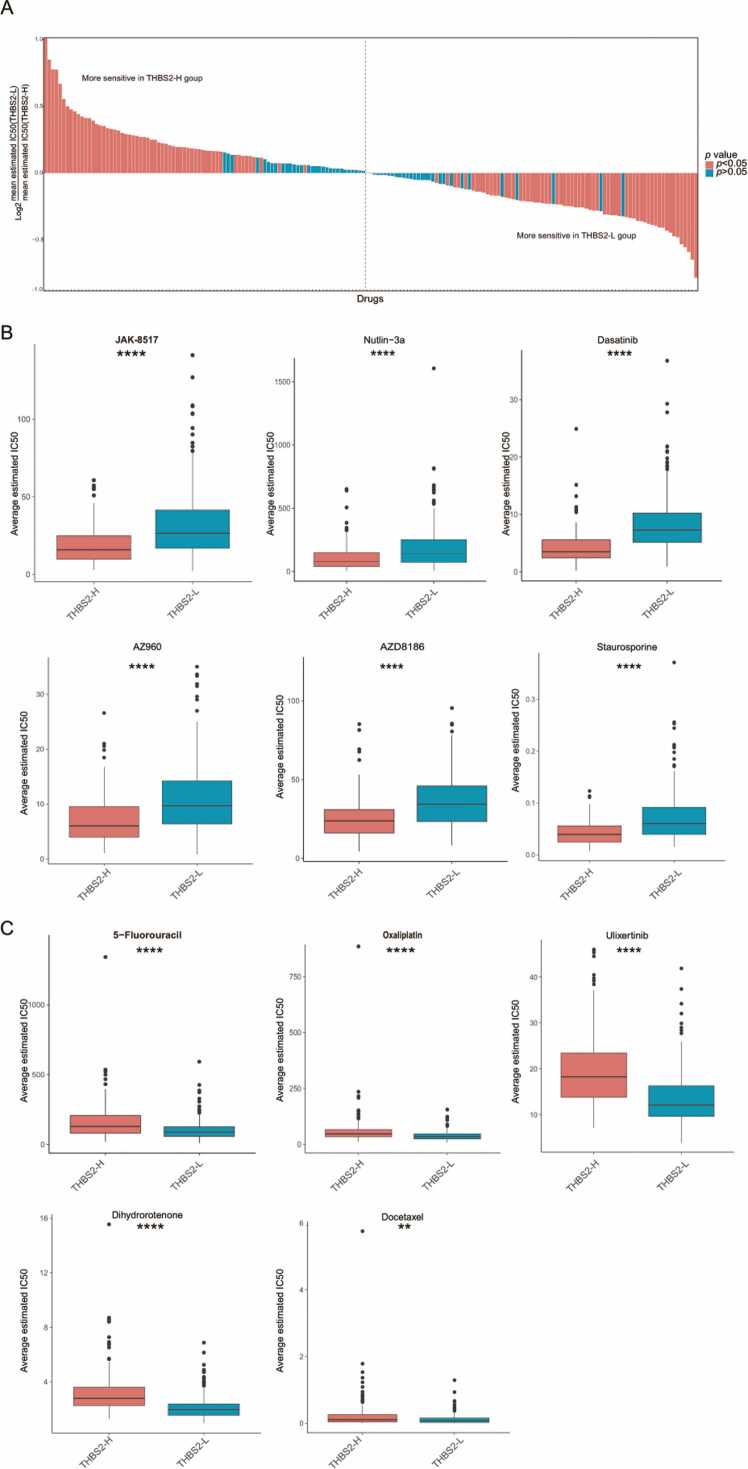
*THBS2* may be associated with drug response in colon cancer patients. **(A)** Significant difference in sensitivity to 179 drugs between THBS2-H and THBS2-L groups. A log_2_FC greater than 0 indicates increased sensitivity of the drug to patients in the THSB2-H group, whereas a value less than 0 indicates increased sensitivity of the drug to patients in the THSB2-L group. **(B)** Box plot showing the six most sensitive drugs in GDSC2 for patients within THBS2-H group. **(C)** Box plot showing the five most sensitive drugs in GDSC2 for patients within THBS2-L group.

Our study demonstrates that *THBS2* is linked to angiogenesis, EMT, immune response, differentiation and function of CAFs, and holds prognostic significance for recurrence-free survival. We continued to investigate the value of *THBS2* in predicting drug response. Initially, we compared the response of patients in the THBS2-H and THBS2-L groups to 179 drugs in GDSC2. The difference in response to drugs between two groups was significant (Fig. 9**A**). Subsequently, we screened patient-specific sensitive drugs for the THBS2-H and THBS2-L groups. Based on the boxplot, patients in the THBS2-H group had lower IC50 values for Dasatinib, Nutlin-3a, JAK-8517, Staurosporine, AZ960, and AZD8186; whereas patients in the THBS2-L group had lower IC50 values for Ulixertinib, Dihydrorotenone, 5-Fluorouracil, Oxaliplatin, and Docetaxel (Fig. 9**B-C**).

## 4 Discussion

Colon cancer remains one of the leading causes of cancer-related deaths globally[47], [48], and its progression is closely linked to the tumor microenvironment [49], [50].Throughout tumor progression, the extracellular matrix of TME undergoes substantial restructuring in its structure and biology, forming ecological niches that foster cancer proliferation, invasion, and metastasis [51]. The elevated expression of matrix proteins characterizes matrix remodeling[6], [8]. Functioning as a link between the ECM and cells, these matrix proteins leverage their diverse functional structures to modulate both intercellular and cell-matrix interactions [8]. As a result, they significantly impact biological processes, including anti-tumor immunity, angiogenesis, and epithelial-mesenchymal transition [5], [8]. Thrombospondins, a family of matrix glycoproteins, plays a crucial role in anti-tumor immunity, angiogenesis, and EMT in tumors such as breast, melanoma, gastric, and liver cancer [4], [16], [42], [52], [53]. Nevertheless, investigations concerning the impact of TSP family members on colon cancer's immune response and EMT have been limited [23], [54], which restricts its clinical application.

In our study, we comprehensively investigated the role of the TSP family in colon cancer using bulk RNA-seq, scRNA-seq and spatial transcriptome data. We found that all five TSP molecules had the potential to predict patients' relapse-free survival, with higher TSPs expression being associated with worse relapse-free survival. Based on GSEA, clusterprofiler enrichment analysis, and correlation analysis, we determined that TSPs were significantly enriched in immune response, EMT, angiogenesis, and lipid metabolism, which have implication for understanding their role in colon cancer progression.

It is vitally important to predict patients at risk of recurrence early. Our multi-cohort survival analysis indicated that the TSPs-H group was characterized by worse RFS. What’s more, compared to the TSPs-L group, the TSPs-H group had higher scores in immune response, epithelial-mesenchymal transition, and angiogenesis. After the validation of ssGSEA, Quantiseq, Timer, Ips, MCP, Epic and Xcell, we found that the immune cell infiltration was indeed higher in the TSPs-H group. At the same time, we found that immunosuppression and immune escape were also present in the TSPs-H group. According to previous studies, TSPs induce an immunosuppressive response. For instance, *THBS1* and *THBS2* bind to the CD47 receptor on the surface of T cells, inducing their apoptosis [55]. In addition to acting as an endogenous suppressor of T cells, *THBS1* also stimulates Tregs to mediate its immunosuppressive effects [56]. Moreover, the upregulation of stromal activity is immunosuppressive and reduces the effectiveness of immunotherapy [39]. Therefore, we speculate that TSPs might inhibit the immune function of T cells, thereby influencing colon cancer progression. Additionally, TSPs might also modify the tumor microenvironment, for example, by promoting angiogenesis and EMT, which could indirectly impact immune function. It is possible that both mechanisms are involved. Further experimental validation of these hypotheses is required.

In order to gain a deeper understanding of how TSPs influence immune function, we believe that determining the cellular origin of TSPs is crucial. Combining the results from datasets GSE16655 (n = 12), GSE188711 (n = 6) and GSE200997 (n = 16), we determined that *THBS1* was mainly expressed in fibroblasts, endothelial cells, and monocyte macrophages; *THBS3* in fibroblasts, tumor cells, and T cells; and *THBS2*, *THBS4*, and *COMP* predominantly in fibroblasts. Our study rigorously reveals that fibroblast is the major source of TSPs in the colon cancer microenvironment. Given the heterogeneity of fibroblast subtypes within the TME [57], direct targeting of all fibroblast subtypes may conversely promote cancer progression [58]. Therefore, it is crucial to further investigate the specific fibroblast subtypes from which TSPs are derived. In our study, all fibroblasts expressed CAFs marker genes (*RUNX1*, *COL1A2*, *VCAN*, *FAP*, *TAGLN* and *ACTA2*), suggesting that the fibroblasts in the TME were CAFs. What’s more, we found that *THBS1* was uniformly expressed across all CAFs subtypes, whereas the expression levels of other TSP molecules were significantly higher in pan-dCAFs compared to other CAFs subtypes. Further cell trajectory analysis revealed differential expression of *THBS2*, *THBS4*, and *COMP* across each CAFs subtype. Among them, *THBS2* and *COMP* may be associated with the conversion of CAFs phenotypes. Previous studies have also found that *THBS2* activates fibroblasts into CAFs [20], [59].

We further explored the role of *THBS2* in the function of CAFs. We found that CAFs communicated most frequently with vascular endothelial cells, monocyte-macrophages, and tumor cells. What's more, compared to *THBS2*^-^ CAFs, *THBS2*^+^ CAFs exhibited stronger communication with other cells. GSEA results also showed that *THBS2*^+^ CAFs were significantly enriched in several pro-cancer pathways including angiogenesis, EMT and glycolysis. In addition, GSVA results indicated that *THBS2*^+^ CAFs exhibited higher scores in hallmark pathways related to matrix remodeling, angiogenesis, cell metabolism, immune response, and common cancer signaling. The TGF-β signaling pathway plays an essential role in the invasion and migration of tumor cells [60]. The Wnt/β-Catenin signaling pathway is a recognized driver of colon cancer [61]. Aberrant activation of the Hedgehog and Notch pathway is associated with colon cancer occurrence and progression [62], [63]. We found that the mean expression levels of receptor-ligand pairs involved in these signaling pathways were higher in the *THBS2*^+^ CAFs. These results were consistent with the previous GSVA findings. It's important to note that our study has not determined whether *THBS2* retains higher expression levels of receptor-ligand pairs involved in the four pathways mentioned above in the absence of *THBS1*. In other words, the observed results might also be attributable to *THBS1*. Studies have shown that CAFs secrete the chemokines SDF-1 and CXCL12 to recruit endothelial progenitor cells and subsequent angiogenesis in tumors [64]. In prostate cancer, researchers find that CAFs secretes CXCL12 and CXCL14 to promote macrophage M2 polarization [65]. *THBS2* causes the death of activated T cells by binding to the CD47 receptor [55]. CAFs express TGF-β1 to suppress the antitumor activity of effector T cells and NK cells [66]. By producing chemokines, CAFs attract and activate immunosuppressive cells such as M2 TAMs, MDSC and Tregs [67]. Our results showed that most T-cell exhaustion marker genes were significantly higher in THBS2-H group. Combining findings from the literature with our results, we speculate that *THBS2* may enhance the immunosuppressive effects of CAFs. Specifically, *THBS2* might first enhance the ability of CAFs to regulate endothelial cells and monocyte-macrophages. Second, it might directly inhibit the cytotoxic activity of T cells. Third, additional mechanisms may also be involved. However, these hypotheses require further validation.

It has been shown that *THBS2* is negatively correlated with the response to immunotherapy [68], prompting us to further investigate the effect of *THBS2* on drug sensitivity. According to our findings, *THBS2* may be associated with chemotherapy drugs response. Patients in the THBS2-H group had lower IC50 values for Dasatinib, Nutlin-3a, JAK-8517, Staurosporine, AZ960, and AZD8186; while patients in the THBS2-L group had lower IC50 values for Ulixertinib, Dihydrorotenone, 5-Fluorouracil, Oxaliplatin, and Docetaxel. Dasatinib is a tyrosine kinase inhibitor that inhibits tumor cell proliferation and invasion [69]. Combined use of Dasatinib and Nutlin-3 can be used to treat chronic B lymphocytic leukemia with p53^wild-type^ and p53^deleted/mutated^ [69]. JAK-8517 is an inhibitor of the JAK/STAT pathway, which induces colon cancer cells apoptosis [70]. 5-Fluorouracil and Oxaliplatin have been approved by the Food and Drug Administration (FDA) for the treatment of colon cancer; A number of clinical studies have demonstrated that docetaxel is effective against breast cancer, colorectal cancer, lung cancer, ovarian cancer, and other types of cancer [71]. Based on the above research, it can be concluded that drugs screened in our study have the potential to treat colon cancer. Additionally, the THBS2-L group exhibited lower IC50 values for 5-fluorouracil and oxaliplatin, which are commonly used in the treatment of colon cancer. This highlights the relevance of assessing *THBS2* expression levels prior to drug treatment.

Of course, this study has several limitations. Firstly, as the TSP family shows low expression in fibroblasts from two other single-cell cohorts (GSE188711 and GSE200997), we only analyzed the association between the TSP family and fibroblast differentiation and function in the GSE166555 cohort (n = 12). Secondly, given THBS2's prominent role in differential expression, correlation analysis, functional enrichment, and cell trajectory analyses, this study primarily focused on exploring the association between *THBS2* and cancer-associated fibroblast function. Additionally, this study found that *THBS2* affects CAFs differentiation and function solely in colon cancer; future research will further explore this molecule's role across various cancers. Lastly, due to funding and platform constraints, this study did not incorporate basic experiments to validate the findings. In future research, we plan to collaborate actively with other researchers to enhance the study's depth and credibility. Nevertheless, our study offers an important theoretical reference for revealing the molecular mechanisms by which TSPs influence colon cancer progression.

In conclusion, our study revealed the role of the TSP family in colon cancer, where this family was closely related to anti-tumour immunity in colon cancer. Notably, *THBS2* was highly associated with the transformation and pro-cancer activity of CAFs. Our findings provide an essential theoretical reference for targeting TSPs to delay colon cancer progression.

## Ethics statement

All data employed in this study were obtained from established public databases, including The Cancer Genome Atlas (TCGA), Gene Expression Omnibus (GEO), Human Protein Atlas (HPA), and University of California Santa Cruz Genome Browser (UCSC). The collection of this data has been duly authorized by the necessary ethical approval bodies. Given these considerations, we are confident that our research adheres to the applicable ethical review standards.

## Funding

This study was supported by the Science and Technology Research Plan Project of Chongqing Education Commission (KJQN202100418), the 10.13039/501100005230Natural Science Foundation of Chongqing (No.cstc2021jcyj-msxm0317), the 10.13039/501100001809National Natural Science Foundation of China (No.82203591), the Medical Science Foundation of the Chengdu Municipal Health Commission (No.2022299).

## CRediT authorship contribution statement

**Ao Tang:** Writing – review & editing, Data curation. **LuYao Tian:** Writing – review & editing, Methodology. **Juan Chen:** Writing – review & editing, Methodology, Funding acquisition. **LiHui Ding:** Writing – review & editing. **mingwei liu:** Writing – review & editing, Writing – original draft, Methodology. **Jing Li:** Writing – review & editing, Writing – original draft, Visualization, Validation, Methodology, Investigation, Formal analysis, Data curation. **Fei Long:** Visualization, Software, Methodology. **Ying Tang:** Writing – review & editing, Software, Methodology.

## Conflict of interest

The authors declare that they have no conflicts of interest.
