# Supplementary material for: Integrating bulk RNA-seq and scRNA-seq analyses revealed the function and clinical value of thrombospondins in colon cancer
Source: Comput Struct Biotechnol J. 2024 May 17;23:2251–66. doi: 10.1016/j.csbj.2024.05.021 (PMC11140486; doi:10.1016/j.csbj.2024.05.021)
Supplement: Supplementary file 1 — Supplementary material [file mmc1.docx]

**Supplementary Figures**

**Figure S1.** Heterogeneity of TSPs expression in colon cancer and prognostic value.

**Figure S2.** Protein expression of TSPs in the human protein atlas (HPA).

**Figure S3.** TSPs was significantly associated with cancer progression-related genes.
**Figure S4.** TSPs was significantly and positively associated with most immune molecules.

**Figure S5.** Stratification of colon cancer patients based on TSPs expression.

**Figure S6.** Differences in tumor microenvironment between TSPs-H and TSPs-L groups.

**Figure S7.** Single-cell analysis localized the cellular origin of the TSP family in the colon cancer microenvironment.

**Figure S8.** Spatial localization of specific markers, cytoTRACE and Slingshot, were employed to assist in determining the source of TSPs and the direction of the cell trajectory, respectively.

**Figure S9.** THBS2 may be associated with enhanced tumor-promoting activity of CAFs.

**Figure S10.** Higher expression levels of receptor-ligand pairs involved in the Notch signaling pathway and Wnt/β-Catenin signaling pathway in THBS2^+^ CAFs compared to THBS2^-^ CAFs.

**
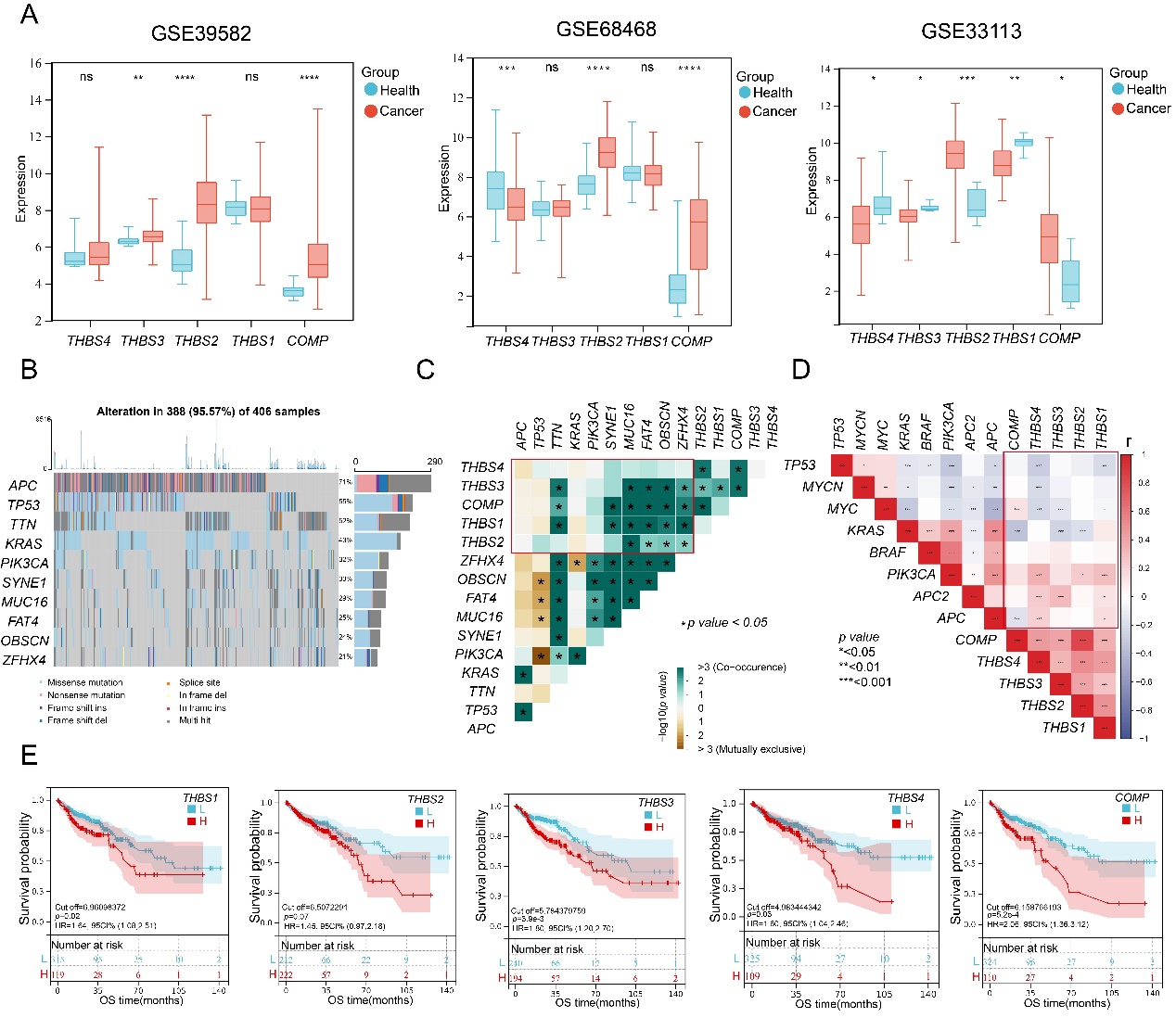
**

**Figure S1.** Heterogeneity of TSPs expression in colon cancer and prognostic value.

**(A)** Differential expression of TSPs in Colon Cancer versus Normal Tissues across datasets GSE39582, GSE68468, and GSE33113. The wilcox test was used to assess the significance of differences between the two groups. ns, no significance, **p* < 0.05, ***p* < 0.01, ****p* < 0.001, *****p* < 0.0001. **(B)** The top 10 mutated genes in colon cancer by R package maftools. **(C)** Co-mutations of TSPs with Top10 mutated genes in colon cancer. **(D)** Co-expression of TSPs with common proto-oncogenes and oncogenes. **(E)**The Kaplan–Meier plot of colon patients based on TSPs expression in the dataset of TCGA-COAD.


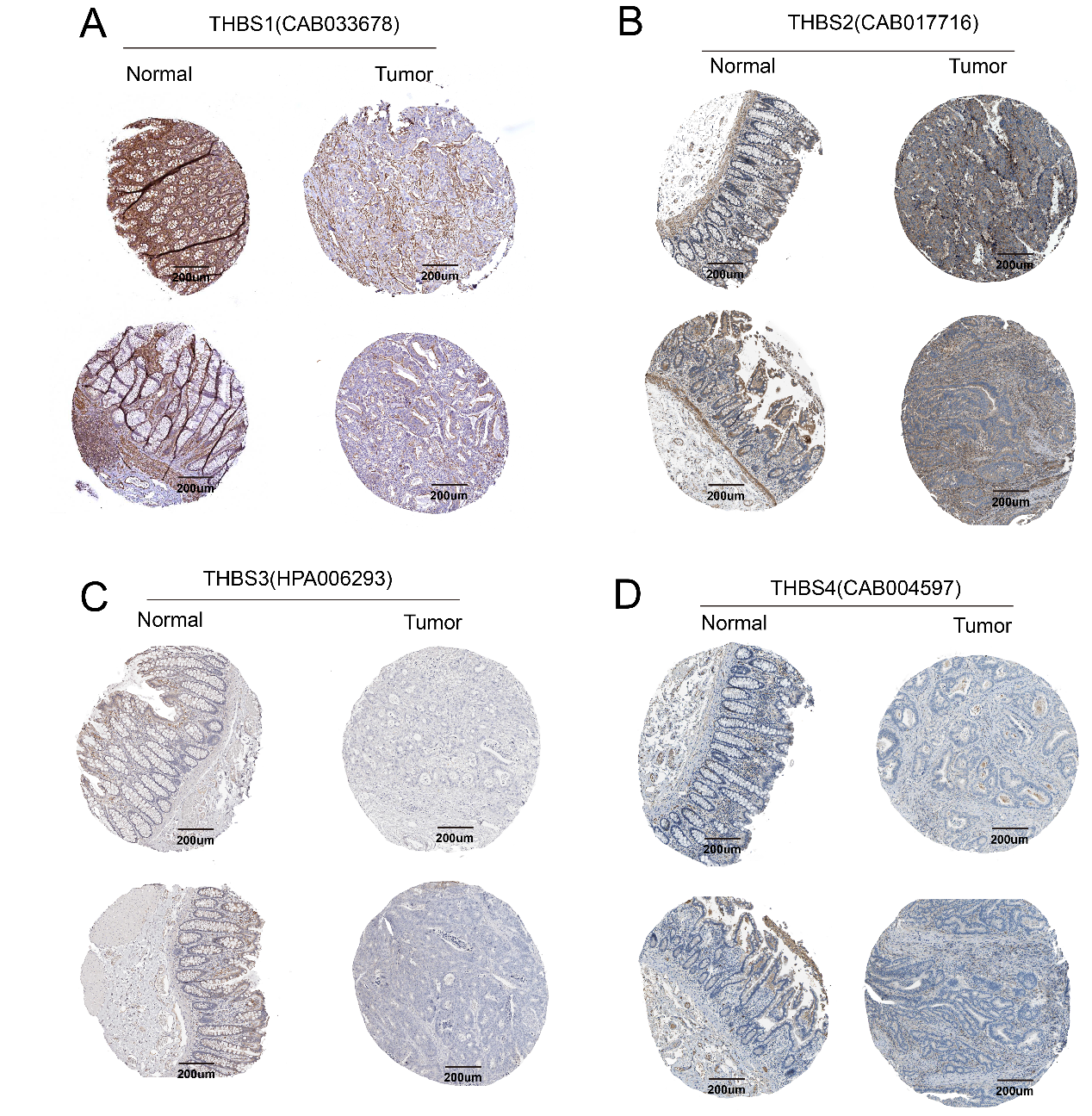


**Figure S2.** Protein expression of TSPs in the human protein atlas (HPA).


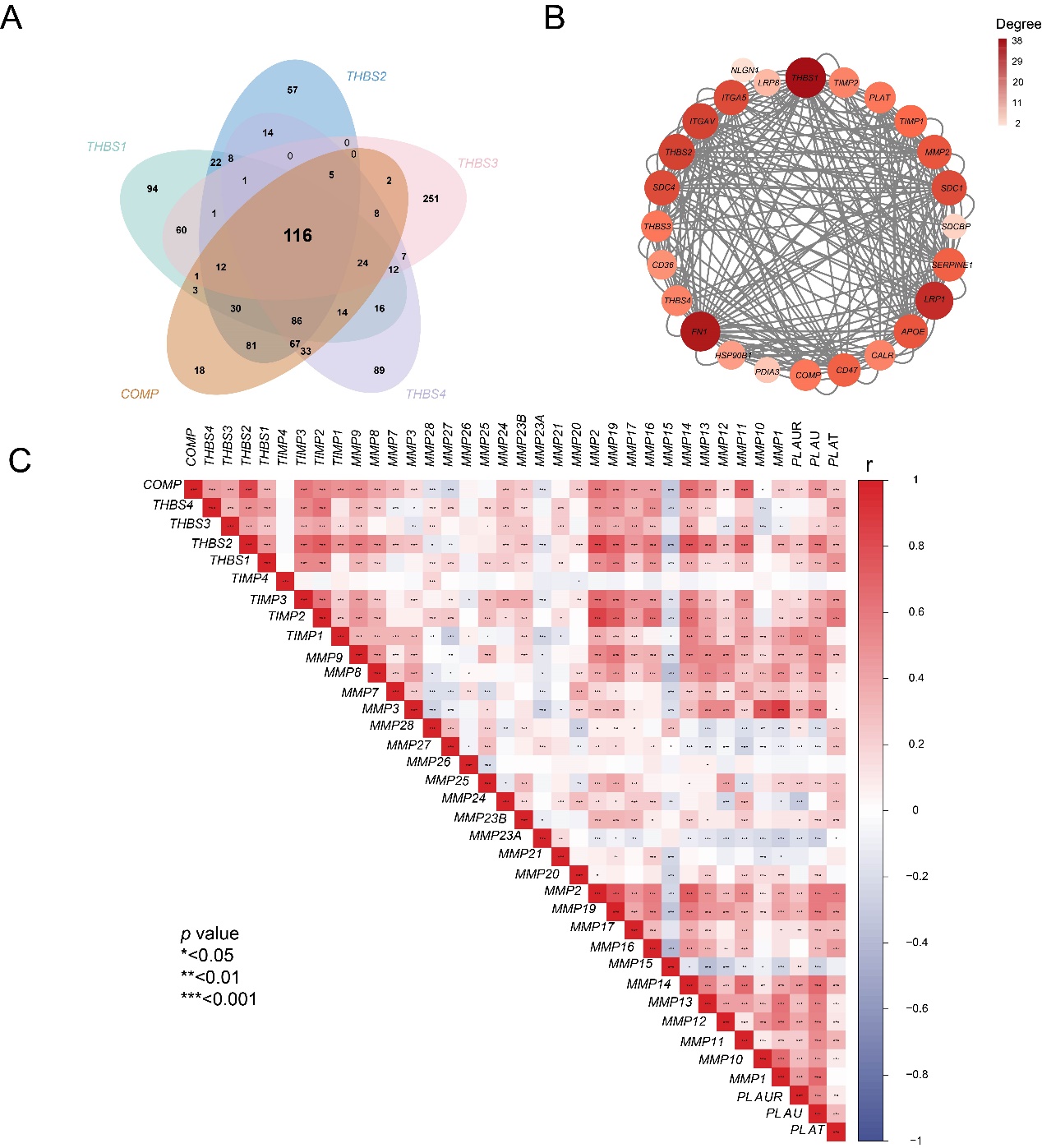


**Figure S3.** TSPs was significantly associated with cancer progression-related genes.

**(A)** The venn plot illustrates the 116 differential genes shared by the TSPs. **(B)** Molecules closely related to TSPs function (rendered by cytoscape). The size and color of the circles in the interaction network were adjusted to reflect the degree of the association. **(C)** Co-expression of TSPs with TIMP, MMP and UPA, box colors are based on correlation coefficients.


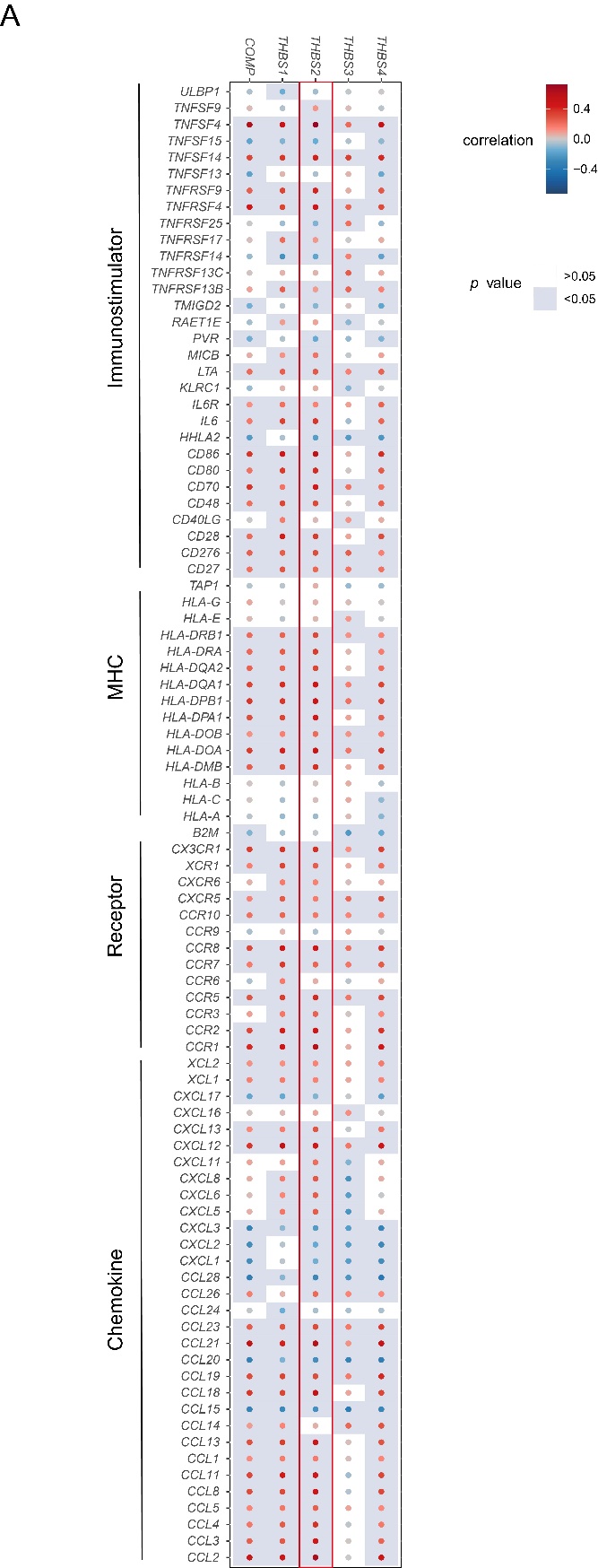


**Figure S4. TSPs was significantly and positively associated with most immune molecules**.


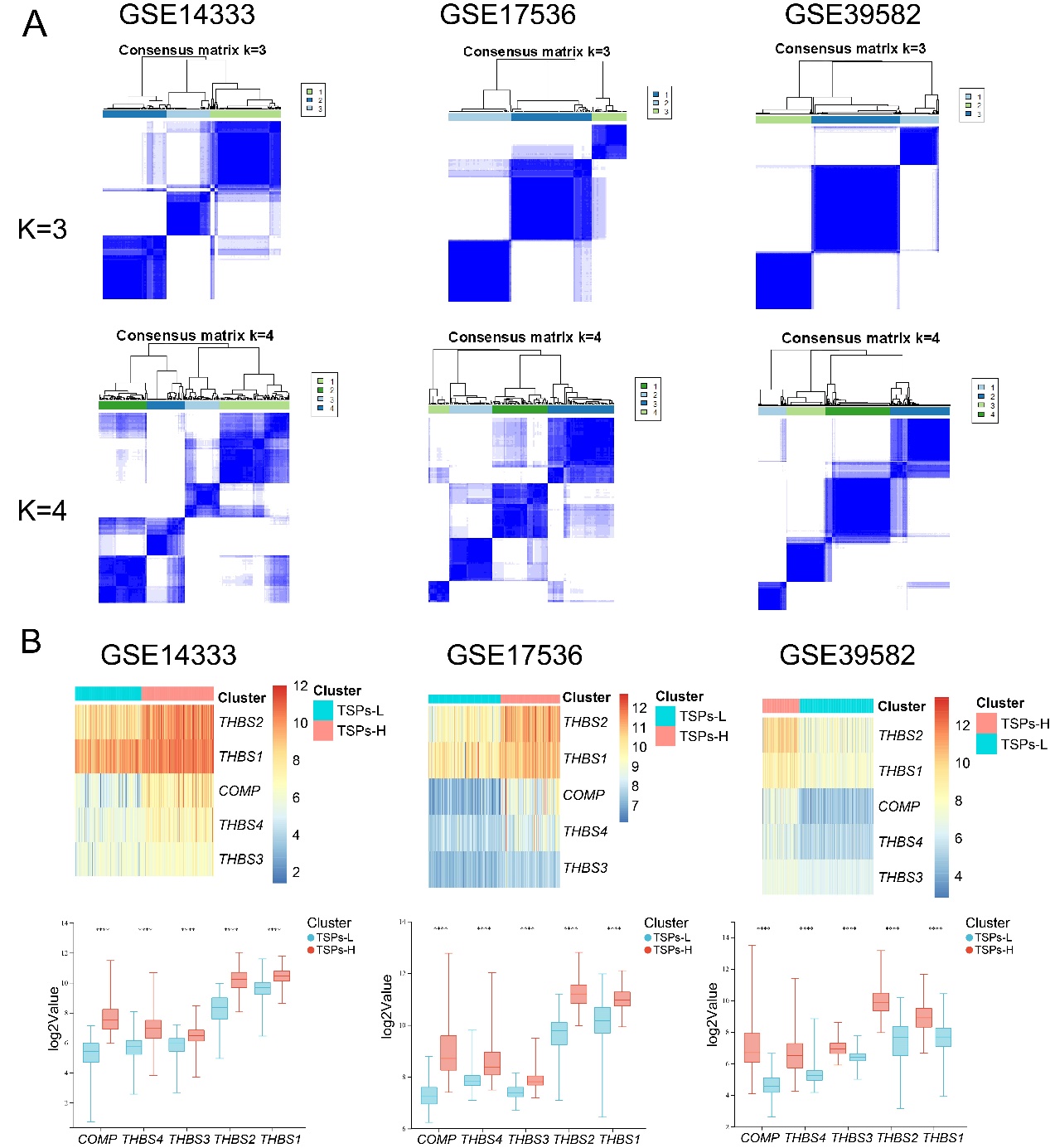


**Figure S5.** Stratification of colon cancer patients based on TSPs expression.

(**A**) Clusters of colon cancer patients in the three GEO cohorts (GSE14333, GSE17536, GSE39582) when k=3 and 4. (**B**) Heatmap and box plot illustrating the differential expression of TSPs in three independent cohorts (TSPs-L vs. TSPs-H)


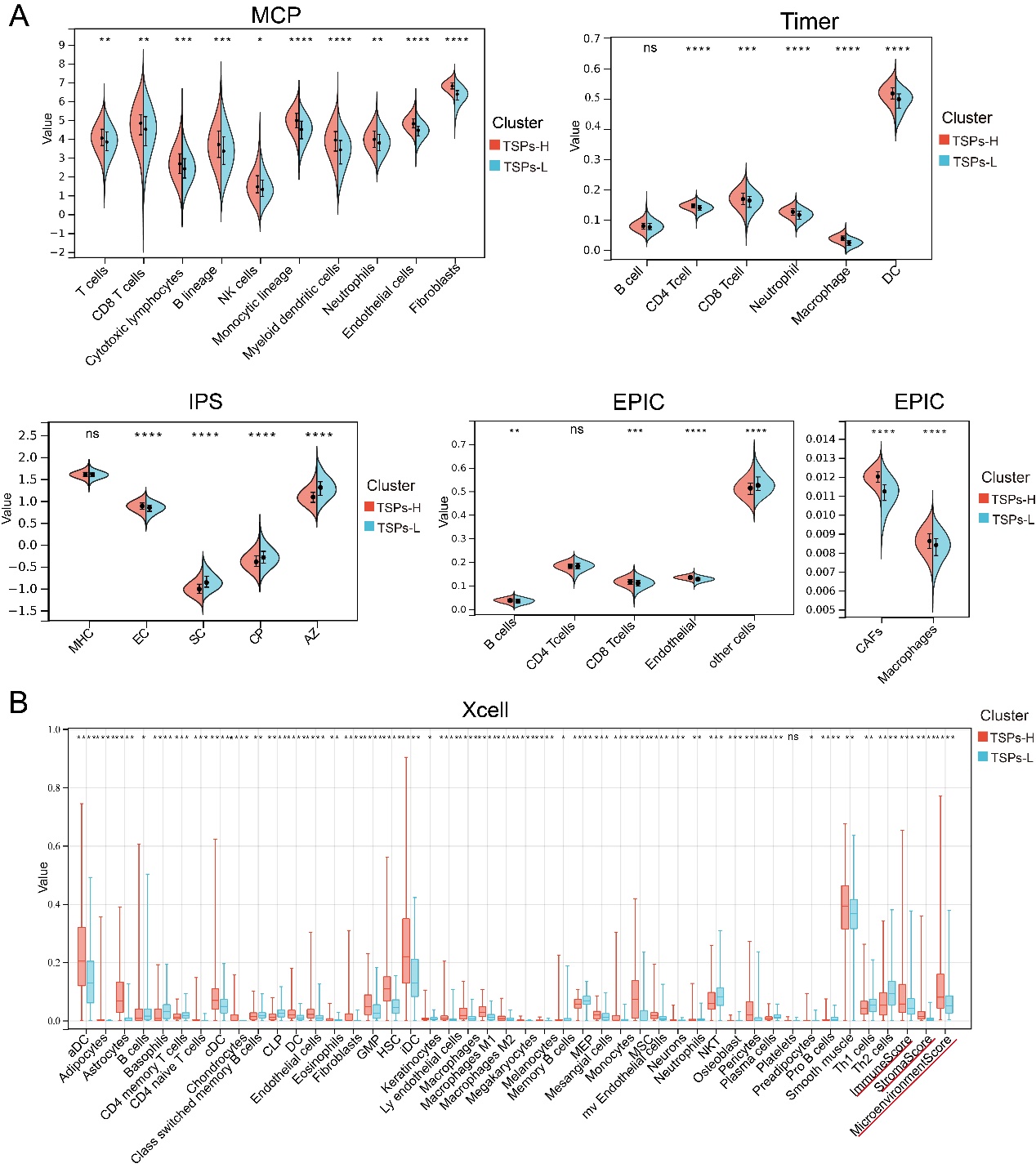


**Figure S6.** Differences in tumor microenvironment between TSPs-H and TSPs-L groups.

**(A)** Assessment of infiltrating cell component in the tumor microenvironment of two clusters of patients by MCP, Timer, IPS, EPIC and **(B)** Xcell.


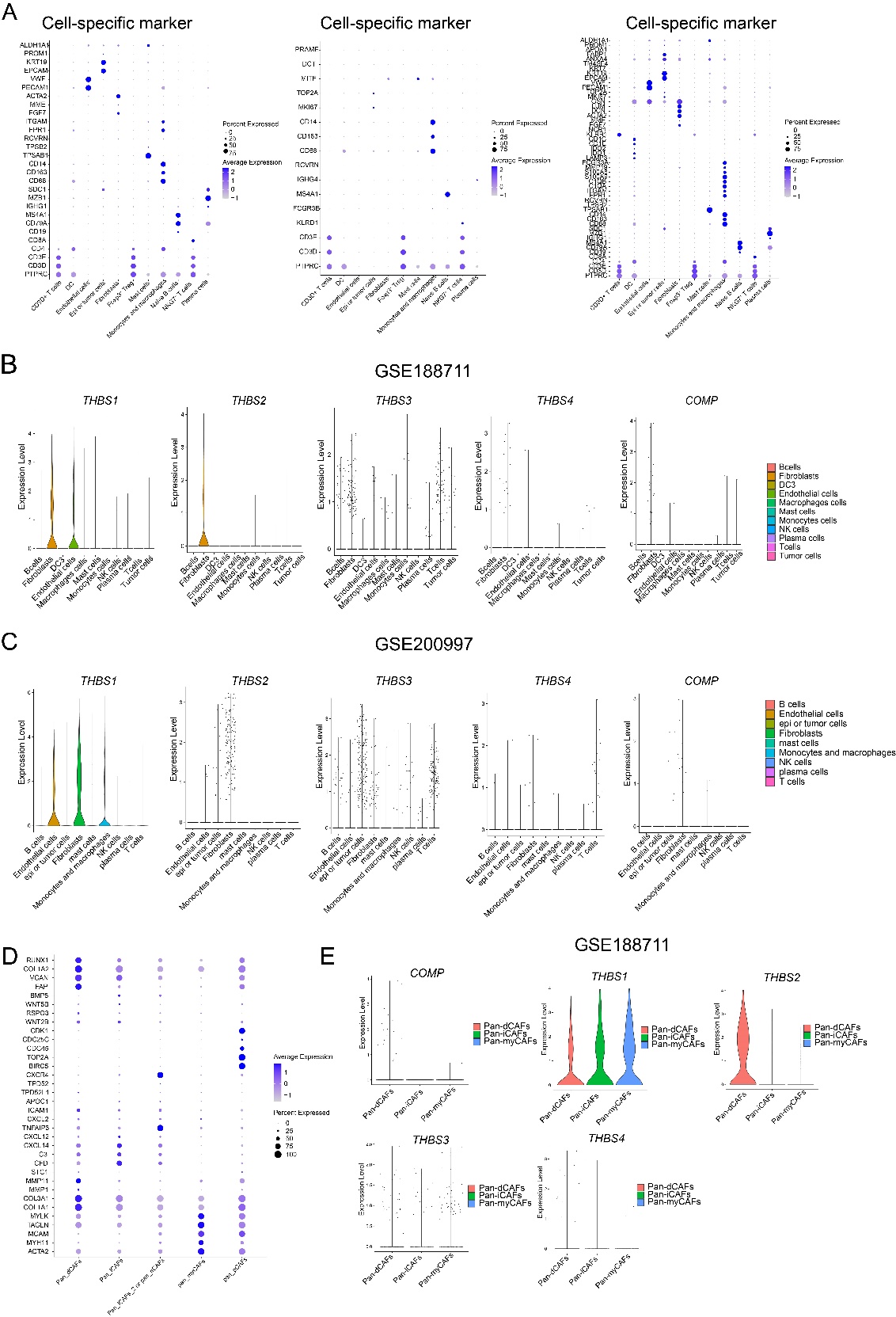


**Figure S7.** Single-cell analysis localized the cellular origin of the TSP family in the colon cancer microenvironment.

**(A)** Expression levels of marker genes in different celltypes. **(B)** vnplot demonstrates the expression of five TSPs molecules in the GSE188711 cohort and the **(C)**GSE20097 cohort in various cell types, with each dot representing a cell. **(D)** Expression levels of CAFs marker genes in different CAFs subtypes. **(E)** vnplot demonstrates the expression of TSPs molecules in CAFs subtypes (GSE188711).


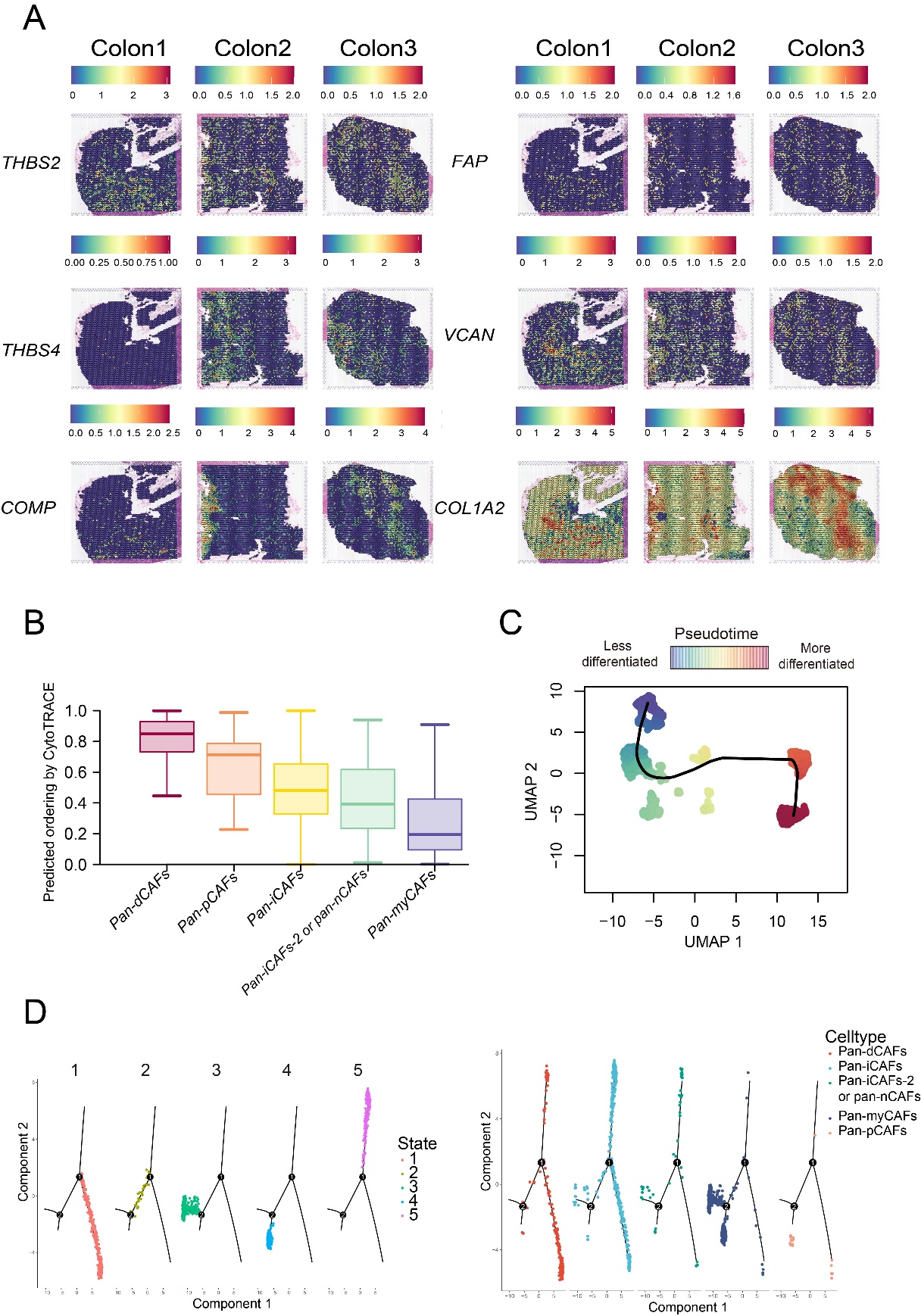


**Figure S8.** Spatial localization of specific markers, cytoTRACE and Slingshot, were employed to assist in determining the source of TSPs and the direction of the cell trajectory, respectively.

**(A)** Spatial localization of *THBS2*, *THBS4*, *COMP* and CAFs marker genes in colon cancer. The redder the spot, the higher the gene expression. **(B)** cytoTRACE scores of five CAFs subtypes. **(C)**Assessment of cell differentiation using Slingshot. **(D)** Distribution of cell groups corresponding to each state in the GSE166555 cohort.


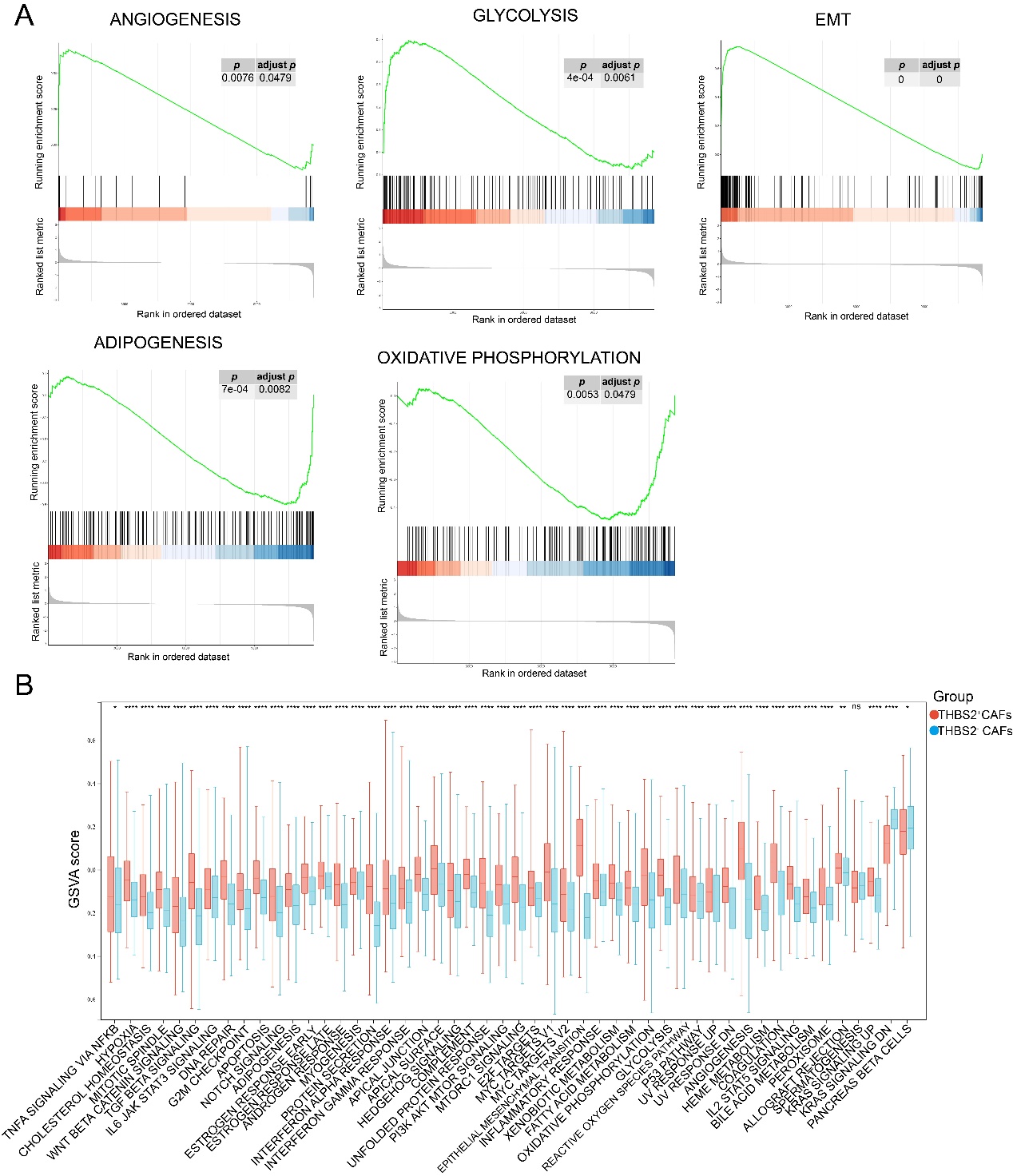


**Figure S9.** THBS2 may be associated with enhanced tumor-promoting activity of CAFs.

**(A)** Pathways significantly enriched in *THBS2*^+^ CAFs compared to *THBS2*^-^ CAFs. **(B)** Differences in hallmark pathway scores between *THBS2*^+^ CAFs and *THBS2*^-^ CAFs groups.


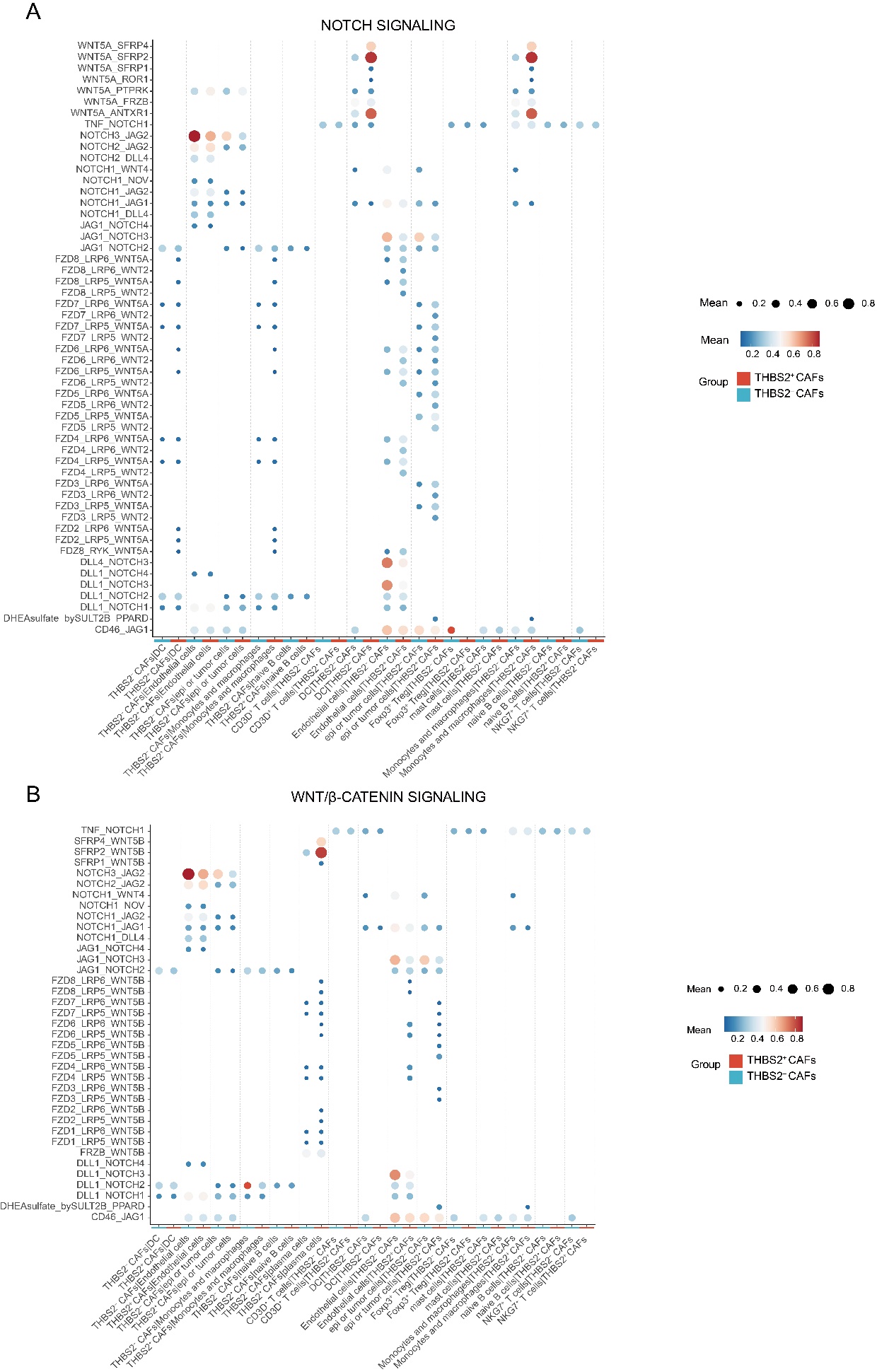


**Figure S10.** Higher expression levels of receptor-ligand pairs involved in the Notch signaling pathway and Wnt/β-Catenin signaling pathway in *THBS2*^+^ CAFs compared to *THBS2*^-^ CAFs.
