# Supplementary material for: Integrating bulk RNA-seq and scRNA-seq analyses revealed the function and clinical value of thrombospondins in colon cancer
Source: Comput Struct Biotechnol J. 2024 May 17;23:2251–66. doi: 10.1016/j.csbj.2024.05.021 (PMC11140486; doi:10.1016/j.csbj.2024.05.021)
Supplement: Supplementary file 2 — Supplementary material [file mmc2.docx]

| **Table S19**. Current Research Status of the TSP Family in the Occurrence and Development of Colon Cancer | | |
| --- | --- | --- |
| **TSP Family** | **Biological Process** | **Reference** |
| *THBS1* | Migration/metastasis | [1-4] |
|  | Angiogenesis | [3, 5-11] |
|  | Immune response | [12] |
|  | Proliferation | [1, 2, 7, 13] |
| *THBS2* | Migration/metastasis | [14-19] |
|  | Angiogenesis | [5] |
|  | Immune response | [20, 21] |
|  | EMT | [15] |
|  | Proliferation | [18] |
| *THBS4* | Migration/metastasis | [22] |
|  | Angiogenesis | [22] |
|  | Proliferation | [22-24] |
| *COMP* | Migration/metastasis | [25-27] |
|  | Immune response | [28] |
|  | EMT | [29] |
|  | Proliferation | [25, 30] |

Notes：EMT，Epithelial-Mesenchymal Transition

**Reference**

[1] Zabrenetzky V, et al., Expression of the extracellular matrix molecule thrombospondin inversely correlates with malignant progression in melanoma, lung and breast carcinoma cell lines*.* *Int J Cancer*, (1994). 191-5. doi:10.1002/ijc.2910590209

[2] Yin Q, et al., MiR-19a enhances cell proliferation, migration, and invasiveness through enhancing lymphangiogenesis by targeting thrombospondin-1 in colorectal cancer*.* *Biochem Cell Biol*, (2019). 731-739. doi:10.1139/bcb-2018-0302

[3] Jia L and D J Waxman, Thrombospondin-1 and pigment epithelium-derived factor enhance responsiveness of KM12 colon tumor to metronomic cyclophosphamide but have disparate effects on tumor metastasis*.* *Cancer Lett*, (2013). 241-9. doi:10.1016/j.canlet.2012.11.055

[4] Radziwon-Balicka A, et al., Mechanisms of platelet-stimulated colon cancer invasion: role of clusterin and thrombospondin 1 in regulation of the P38MAPK-MMP-9 pathway*.* *Carcinogenesis*, (2014). 324-32. doi:10.1093/carcin/bgt332

[5] Tsuchida T, et al., Expression of the thrombospondin 1 receptor CD36 is correlated with decreased stromal vascularisation in colon cancer*.* *Int J Oncol*, (1999). 47-51.

[6] Jo W S, et al., Wnt signaling can repress thrombospondin-1 expression in colonic tumorigenesis*.* *Cancer Biol Ther*, (2005). 1361-6. doi:10.4161/cbt.4.12.2201

[7] Lopez-Dee Z P, et al., Thrombospondin-1 in a Murine Model of Colorectal Carcinogenesis*.* *PLoS One*, (2015). e0139918. doi:10.1371/journal.pone.0139918

[8] Maeda K, et al., Expression of vascular endothelial growth factor and thrombospondin-1 in colorectal carcinoma*.* *Int J Mol Med*, (2000). 373-8. doi:10.3892/ijmm.5.4.373

[9] Miyanaga K, et al., Expression and role of thrombospondin-1 in colorectal cancer*.* *Anticancer Res*, (2002). 3941-8.

[10] Allegrini G, et al., Thrombospondin-1 plus irinotecan: a novel antiangiogenic-chemotherapeutic combination that inhibits the growth of advanced human colon tumor xenografts in mice*.* *Cancer Chemother Pharmacol*, (2004). 261-6. doi:10.1007/s00280-003-0712-y

[11] Kim M S, et al., Extracellular Matrix Biomarkers in Colorectal Cancer*.* *Int J Mol Sci*, (2021). doi:10.3390/ijms22179185

[12] Omatsu M, et al., THBS1-producing tumor-infiltrating monocyte-like cells contribute to immunosuppression and metastasis in colorectal cancer*.* *Nat Commun*, (2023). 5534. doi:10.1038/s41467-023-41095-y

[13] Teraoku H, et al., Role of thrombospondin-1 expression in colorectal liver metastasis and its molecular mechanism*.* *J Hepatobiliary Pancreat Sci*, (2016). 565-73. doi:10.1002/jhbp.376

[14] He Z, et al., Identification of BGN and THBS2 as metastasis-specific biomarkers and poor survival key regulators in human colon cancer by integrated analysis*.* *Clin Transl Med*, (2022). e973. doi:10.1002/ctm2.973

[15] Zhang C, et al., The Integrative Analysis of Thrombospondin Family Genes in Pan-Cancer Reveals that THBS2 Facilitates Gastrointestinal Cancer Metastasis*.* *J Oncol*, (2021). 4405491. doi:10.1155/2021/4405491

[16] Qu H L, et al., THBS2 promotes cell migration and invasion in colorectal cancer via modulating Wnt/beta-catenin signaling pathway*.* *Kaohsiung J Med Sci*, (2022). 469-478. doi:10.1002/kjm2.12528

[17] Yoshida Y, et al., Expression of angiostatic factors in colorectal cancer*.* *Int J Oncol*, (1999). 1221-5. doi:10.3892/ijo.15.6.1221

[18] Wang X, et al., THBS2 is a Potential Prognostic Biomarker in Colorectal Cancer*.* *Sci Rep*, (2016). 33366. doi:10.1038/srep33366

[19] Jin Z, et al., Type 3 Repeats of Thrombospondin-2 Increases Metastasis in Mouse Colorectal Cancer CT-26 Cells*.* *Chonnam Medical Journal*, (2010). doi:10.4068/cmj.2010.46.1.7

[20] Liu Y, et al., Systematic analysis of integrated bioinformatics to identify upregulated THBS2 expression in colorectal cancer cells inhibiting tumour immunity through the HIF1A/Lactic Acid/GPR132 pathway*.* *Cancer Cell Int*, (2023). 253. doi:10.1186/s12935-023-03103-5

[21] Deng B, X P Liu, and X Wang, Prognostic and Immunological Role of THBS2 in Colorectal cancer*.* *Biomed Res Int*, (2021). 1124985. doi:10.1155/2021/1124985

[22] Kim M S, et al., Potential Role of PDGFRbeta-Associated THBS4 in Colorectal Cancer Development*.* *Cancers (Basel)*, (2020). doi:10.3390/cancers12092533

[23] Stenina-Adognravi O, S Muppala, and J Gajeton, Thrombospondins and remodeling of the tumor microenvironment*.* *Vessel Plus*, (2018). doi:10.20517/2574-1209.2018.40

[24] Greco S A, et al., Thrombospondin-4 is a putative tumour-suppressor gene in colorectal cancer that exhibits age-related methylation*.* *BMC Cancer*, (2010). 494. doi:10.1186/1471-2407-10-494

[25] Guo B, et al., Cartilage oligomeric matrix protein acts as a molecular biomarker in multiple cancer types*.* *Clin Transl Oncol*, (2023). 535-554. doi:10.1007/s12094-022-02968-8

[26] Nfonsam V N, et al., Increasing Incidence of Colon Cancer in the Young: Assessing the Tumor Biology*.* *J Am Coll Surg*, (2019). 79-90. doi:10.1016/j.jamcollsurg.2019.03.022

[27] Zhong W, et al., Cartilage Oligomeric Matrix Protein promotes epithelial-mesenchymal transition by interacting with Transgelin in Colorectal Cancer*.* *Theranostics*, (2020). 8790-8806. doi:10.7150/thno.44456

[28] Ma H, et al., The Cancer-Associated Fibroblasts-Related Gene COMP Is a Novel Predictor for Prognosis and Immunotherapy Efficacy and Is Correlated with M2 Macrophage Infiltration in Colon Cancer*.* *Biomolecules*, (2022). doi:10.3390/biom13010062

[29] Nfonsam V N, et al., COMP Gene Coexpresses With EMT Genes and Is Associated With Poor Survival in Colon Cancer Patients*.* *J Surg Res*, (2019). 297-303. doi:10.1016/j.jss.2018.08.021

[30] Liu T T, et al., Cartilage oligomeric matrix protein is a prognostic factor and biomarker of colon cancer and promotes cell proliferation by activating the Akt pathway*.* *J Cancer Res Clin Oncol*, (2018). 1049-1063. doi:10.1007/s00432-018-2626-4
