# Supplementary material for: Integrating bulk RNA-seq and scRNA-seq analyses revealed the function and clinical value of thrombospondins in colon cancer
Source: Comput Struct Biotechnol J. 2024 May 17;23:2251–66. doi: 10.1016/j.csbj.2024.05.021 (PMC11140486; doi:10.1016/j.csbj.2024.05.021)
Supplement: Supplementary file 3 — Supplementary material [file mmc3.docx]

**Supplementary Material I**

**Integrating bulk RNA-seq and scRNA-seq analyses revealed the function and clinical value of thrombospondins in colon cancer**

Jing Li^a,†^, Ying Tang^a,b,†^, Fei Long^c,d^, Luyao Tian^a^, Ao Tang^a^, LiHui Ding^a^, Juan Chen^a,*^, Mingwei Liu^a,*^

^a^Key Laboratory of Clinical Laboratory Diagnostics, College of Laboratory Medicine, Chongqing Medical University, Chongqing 400046, China.

^b^Medical Laboratory, People's Hospital of Qingbaijiang District, Chengdu, 61300, China.

^c^Department of Laboratory Medicine, Zhongnan Hospital of Wuhan University, Wuhan, China.

^d^Center for Single-Cell Omics and Tumor Liquid Biopsy, Zhongnan Hospital of Wuhan University, Wuhan, China.

**^*^Correspondence,**

Mingwei Liu, [liumingwei@cqmu.edu.cn;](mailto:liumingwei@cqmu.edu.cn;) Juan Chen, [cladchen@cqmu.edu.cn](mailto:cladchen@cqmu.edu.cn)

†, Authors contributed equally to this work.

**Material and methods**

The workflow of this study, illustrated in **Fig.1**, includes three main Components: exploring the functional roles and stratification value of TSPs, investigating the differences in the tumor microenvironment (TME) between two clusters, and further dissecting the effects of TSPs on the TME using single-cell analysis. Detailed descriptions of each component are provided below.

**1. Data Acquisition and Processing**

The TCGAbiolinks was used to download colon cancer read count sequencing data and related clinical information from TCGA (https://portal.gdc.cancer.gov) for training purposes. The gene expression matrix of GSE39582, GSE68468 and GSE33113 were obtained from GEO ([https://www.ncbi.nlm.nih.gov/geo](https://www.ncbi.nlm.nih.gov/geo/)). What’s more, the microarray datasets GSE14333, GSE17536 and GSE39582, which contain disease-free survival or recurrence-free survival (DFS or RFS) information, were used to verify the accuracy of TSPs in predicting colon cancer recurrence-free survival. Using publicly available spatial transcriptome data, we identified the distribution of TSP family genes and CAFs marker genes in colon cancer^[1]^. We normalized the data using sctransform and utilized the first 20 principal Components with a resolution of 0.8 for clustering.

**2. Copy number, mutation and protein expression analysis**

The somatic mutation profile of TCGA-COAD was identified using MuTect2 and obtained from UCSC Xena (https://xenabrowser.net). We visualized gene mutations using the oncoplot function of the maftools package, and examined the co-mutation and co-expression patterns between TSPs and other genes using the corrplot package. Additionally, we analyzed TSPs copy number variation using GenePattern (https://www.genepattern.org) and visualized the results with Lollipop charts. Using immunohistochemical images obtained from the HPA database (https://www.proteinatlas.org), we assessed the levels of TSPs proteins in colon cancer tissues. To evaluate the impact of TSPs expression dysregulation on patient prognosis, we constructed Kaplan-Meier survival curves and employed the surv-cutpoint algorithm from the survminer package to determine the optimal cutoff values.

**3. Enrichment analysis and correlation analysis**

Using Gene Set Enrichment Analysis (GSEA), we evaluated the biological functions affected by TSPs and displayed them with heatmap. The specific workflow is as follows: First, based on the median expression of each TSP molecule, 455 colon cancer patients in the TCGA-COAD cohort were grouped into high and low expression groups (namely THBS1-H/L, THBS2-H/L, THBS3-H/L, THBS4-H/L, COMP-H/L). Second, we used the edgeR package to perform differential expression analysis for each high and low expression group to obtain gene lists of differentially expressed genes associated with each TSP molecule (**Table S1-S5**). Next, hallmark gene sets were downloaded from the MSigDB database (http://www.gsea-msigdb.org/gsea/msigdb/index.jsp) to serve as the background gene sets. With these, GSEA analyses were conducted using the differentially expressed gene lists from each TSP molecule to identify significantly impacted functional pathways (|*NES*| >1, *P.adjust* < 0.05, and *Q* < 0.05). Finally, we presented the significantly enriched functional pathways of the TSP family using heatmaps. The values in the heatmap represent the Normalized Enrichment Score (NES), with positive values indicating upregulated pathway score and negative values indicating downregulated score. To verify the results of the GSEA, Venn diagrams^[2]^was used to identify genes with TSPs co-differences (n = 116). Subsequently, the clusterprofiler R package was employed to perform GO and KEGG enrichment analyses. Additionally, molecules closely related to TSP functions were identified in the STRING database (https://cn.string-db.org). The corrplot R package was utilized to analyze the correlations between TSPs and EMT marker genes, immune cells and molecules, matrix metalloproteinases and their inhibitors, tissue-type and urokinase-type plasminogen activators. Genes associated with EMT were obtained from the MsigDB database, immune cells were identified using ssGSEA, and immune molecules were derived from a previous study^[3]^.

**4. Identification of molecular clusters based on TSPs expression**

Using the “ConsensusClusterPlus” package, 1000 iterations were performed with a subsampling ratio of 0.8. The datasets GSE14333, GSE17536, and GSE39582 were used as validation sets to demonstrate the stratification value of TSPs. We compared TSPs expression and clinical information between two clusters using the pheatmap R package. The Wilcoxon rank-sum test was used to determine whether the differences in TSPs expression between the two clusters were statistically significant.

**5. Differences in tumor microenvironment between two clusters**

The HallMark gene sets were retrieved from the Msigdb database and used as the background gene set for Gene Set Variation Analysis (GSVA). The pathways activity of each patient was quantified using R package GSVA. The first 31 differentially expressed channels in the heat map all meet *FDR* < 0.05. CIBERSORT^[4]^ and ssGSEA algorithm^[5]^were used to quantify the abundance of immune cell. To confirm differences in immune cell composition between patients in the TSPs-H and TSPs-L groups, we used the IOBR package^[6]^, which integrates eight commonly used methods: CIBERSORT, TIMER, xCell, MCPcounter, ESITMATE, EPIC, IPS, quantTIseq. The Tumor Immune Dysfunction and Exclusion (TIDE) analysis was performed using the online platform (http://tide.dfci.harvard.edu) to evaluate immune escape in each colon cancer patient. The higher the TIDE score, the greater the likelihood of immune escape and the lower the chance of benefiting from immunotherapy. To characterize T cell killing activity, we obtained T cell exhaustion marker genes from previous literature^[7, 8]^and compared their expression between TSPs-H and TSPs-L groups.

**6. Single cell RNA-seq analysis of colon cancer**

The study included three scRNA-seq datasets, GSE166555^[9]^, GSE188711^[10]^ and GSE200997^[11]^, to investigate the origin of TSPs in the TME. The scRNA-seq expression matrix was processed with R package Seurat. To filter out low-quality cells, only cells with more than 300 and fewer than 5000 transcripts per cell (fewer than 3000 for GSE188711) and with transcripts expressed in more than 3 cells; additionally, cells with less than 20% mitochondrial reads (less than 15% for GSE188711) were included in the analysis. The gene expression data was normalized and the 2000 most highly variable genes (HVG) was identified using the FindVariableFeatures function; Principal component Analysis (PCA) was conducted using the RunPCA function, and linear dimensionality was reduced using the first 40 principal components. The Harmony R package was utilized to correct for batch effects. The RunUMAP functions performed nonlinear reduction of dimensionality (*DIM* = 1 : 40). We identified individual cell clusters based on known cell-specific marker genes from literature or CellMarker database (**FigS7A**) ^[12-15]^.

We defined fibroblast subtypes based on fibroblast marker genes from previous literature^[16, 17]^. Specifically, pan-myCAFs exhibited high expression levels of smooth muscle contraction genes (*ACTA2*, *MYH11*, *TAGLN*, *MYLK*) and cell adhesion and signal transduction genes (*MCAM*). Pan-dCAFs were characterized by high expression levels of collagen remodeling genes (*COL1A1*, *COL3A1*, *MMP1*, *MMP11*) and genes involved in calcium-phosphorus homeostasis and cell proliferation (*STC1*). Pan-iCAFs showed elevated levels of immune response genes with complement system (*CFD*, *C3*) and chemokines (*CXCL14*, *CXCL12*). Pan-iCAFs-2 were marked by high expression levels of adhesion (*ICAM1*) and immune response genes (*CXCL2*, *TNFAIP3*). Pan-nCAFs had elevated expression of genes associated with lipid metabolism (*APOC1*), cell proliferation, and apoptosis (*TPD52L1*, *TPD52*), along with immune regulation (*CXCR4*). Pan-pCAFs were characterized by genes linked to cell cycle regulation (*CDC25C*, *CDK1*). The optimal resolution for clustering was achieved by combing FindClusters, clustree, and rogue^[18]^. Fibroblasts extracted from the GSE166555 cohort were further classified into five types: pan-dCAFs, pan-iCAFs, pan-iCAFs-2 or pan-nCAFs, pan-myCAFs, and pan-pCAFs. Similarly, fibroblasts from the GSE188711 cohort were classified into three types: pan-dCAFs, pan-iCAFs and pan-myCAFs. We use the Monocle2 algorithm to analyse the pseudo-time trajectory of CAFs and determine the start and end points of this cell trajectory by means of cytoTRACE^[19]^ and slingshot^[20]^. The FindAllMarkers function was used to identify the top 10 differentially expressed genes (DEGs) for each CAFs subtype ( *P* < 0.01, |log_2_fc| > 0.25). The top 50 genes with the greatest change in expression over the proposed time were identified using the differentialGeneTest function. We applied BEAM to screen for genes closely related to point one branch, with a significance level of *Q* < 0.01, and examined the correlation between TSPs and branch genes using the corrplot R package.

**7. Analysis of cell communication strength and signaling pathway activity scores**

We examined the effect of *THBS2* on the communication between CAFs and other cells using CellPhoneDB, a repository of ligands, receptors, and their interactions^[21]^. We divided CAFs into two groups: *THBS2*^+^ CAFs and *THBS2*^-^ CAFs, with the former expressing *THBS2* and the latter not. Significant cell communication were calculated based on normalized cell matrix achieved by seurat (*P* < 0.05). The number of receptor-ligand pairs between various cell types was visualized using pheatmap (*P* < 0.05). We also compared gene expression of T cell exhaustion markers^[7, 8]^ between the THBS2-H and THBS2-L groups in order to demonstrate the potential immunosuppressive effect of *THBS2*. Furthermore, we utilized dotplot to compare the mean expression levels of receptor-ligand pairs involved in four cancer signaling pathways (Hedgehog signaling pathway, TGF-β signaling pathway, and Notch signaling pathway plus Wnt/β-Catenin signaling pathway) between *THBS2*^+^ and *THBS2*^-^ CAFs. Additionally, the FindMarkers function was used to identify differentially expressed genes (DEGs), followed by GSEA to analyze the biological processes significantly enriched by these genes (*P* < 0.05). Finally, we compared Hallmark pathway activity score between *THBS2*^+^ and *THBS2*^-^ CAFs using GSVA.

**8. Drug sensitivity analyses**

Using the R package oncoPredict, we predicted the response of TCGA patients to 198 drugs from GDSC2. The expression matrix and drug sensitivity matrix of 809 cell lines were obtained from osf.io (https://osf.io/c6tfx). Before performing KNN imputation, drugs and samples with more than 20% missing data were eliminated. Based on the median expression of *THBS2*, colon cancer patients were divided into THBS2-H and THBS2-L groups. Drugs with *P* < 0.05 and |log_2_fc| > 0.5 were sensitive drugs. Theoretically, the lower the IC50 value, the more sensitive the patient was.

**9. Statistical analyses**

R software 4.0.2 was conducted for statistical analysis in this study. Between-group differences and survival analysis were partly conducted using SangerBox (https://www.sangerbox.com).The wilcoxon rank-sum test and kruskal-wallis rank sum test were used to estimate the differences between clusters. Survival curves were plotted using the Kaplan-Meier method, and survival differences were evaluated with the log-rank test. Correlation analysis were performed using the spearman correlation test. The tools and sources involved are listed in **Table S6**.

**10. Webserver development**

A webserver presenting the main findings of this study is available, which includes detailed information on all cohorts and tools used, results, and appendices. The webserver can be accessed at http://tsp.liumwei.org/Col.Cancer.

**Reference**

[1] Wu Y, et al., Spatiotemporal Immune Landscape of Colorectal Cancer Liver Metastasis at Single-Cell Level*.* *Cancer Discov*, (2022). 134-153. doi:10.1158/2159-8290.CD-21-0316

[2] Bardou P, et al., jvenn: an interactive Venn diagram viewer*.* *BMC Bioinformatics*, (2014). 293. doi:10.1186/1471-2105-15-293

[3] Hu J, et al., Siglec15 shapes a non-inflamed tumor microenvironment and predicts the molecular subtype in bladder cancer*.* *Theranostics*, (2021). 3089-3108. doi:10.7150/thno.53649

[4] Newman A M, et al., Robust enumeration of cell subsets from tissue expression profiles*.* *Nat Methods*, (2015). 453-7. doi:10.1038/nmeth.3337

[5] Subramanian A, et al., Gene set enrichment analysis: a knowledge-based approach for interpreting genome-wide expression profiles*.* *Proc Natl Acad Sci U S A*, (2005). 15545-50. doi:10.1073/pnas.0506580102

[6] Zeng D, et al., IOBR: Multi-Omics Immuno-Oncology Biological Research to Decode Tumor Microenvironment and Signatures*.* *Front Immunol*, (2021). 687975. doi:10.3389/fimmu.2021.687975

[7] Chen D, et al., Identification and Characterization of Robust Hepatocellular Carcinoma Prognostic Subtypes Based on an Integrative Metabolite-Protein Interaction Network*.* *Adv Sci (Weinh)*, (2021). e2100311. doi:10.1002/advs.202100311

[8] Zhang Z, et al., Pan-cancer landscape of T-cell exhaustion heterogeneity within the tumor microenvironment revealed a progressive roadmap of hierarchical dysfunction associated with prognosis and therapeutic efficacy*.* *EBioMedicine*, (2022). 104207. doi:10.1016/j.ebiom.2022.104207

[9] Uhlitz F, et al., Mitogen-activated protein kinase activity drives cell trajectories in colorectal cancer*.* *EMBO Mol Med*, (2021). e14123. doi:10.15252/emmm.202114123

[10] Guo W, et al., Resolving the difference between left-sided and right-sided colorectal cancer by single-cell sequencing*.* *JCI Insight*, (2022). doi:10.1172/jci.insight.152616

[11] Khaliq A M, et al., Refining colorectal cancer classification and clinical stratification through a single-cell atlas*.* *Genome Biol*, (2022). 113. doi:10.1186/s13059-022-02677-z

[12] Zhang L, et al., Single-Cell Analyses Inform Mechanisms of Myeloid-Targeted Therapies in Colon Cancer*.* *Cell*, (2020). 442-459 e29. doi:10.1016/j.cell.2020.03.048

[13] Wang F, et al., Single-cell and spatial transcriptome analysis reveals the cellular heterogeneity of liver metastatic colorectal cancer*.* *Sci Adv*, (2023). eadf5464. doi:10.1126/sciadv.adf5464

[14] Hu J, et al., Single-Cell Transcriptome Analysis Reveals Intratumoral Heterogeneity in ccRCC, which Results in Different Clinical Outcomes*.* *Mol Ther*, (2020). 1658-1672. doi:10.1016/j.ymthe.2020.04.023

[15] Zhang X, et al., CellMarker: a manually curated resource of cell markers in human and mouse*.* *Nucleic Acids Res*, (2019). D721-D728. doi:10.1093/nar/gky900

[16] Galbo P M, Jr., X Zang, and D Zheng, Molecular Features of Cancer-associated Fibroblast Subtypes and their Implication on Cancer Pathogenesis, Prognosis, and Immunotherapy Resistance*.* *Clin Cancer Res*, (2021). 2636-2647. doi:10.1158/1078-0432.CCR-20-4226

[17] Becker W R, et al., Single-cell analyses define a continuum of cell state and composition changes in the malignant transformation of polyps to colorectal cancer*.* *Nat Genet*, (2022). 985-995. doi:10.1038/s41588-022-01088-x

[18] Liu B, et al., An entropy-based metric for assessing the purity of single cell populations*.* *Nat Commun*, (2020). 3155. doi:10.1038/s41467-020-16904-3

[19] Gulati G S, et al., Single-cell transcriptional diversity is a hallmark of developmental potential*.* *Science*, (2020). 405-411. doi:10.1126/science.aax0249

[20] Street K, et al., Slingshot: cell lineage and pseudotime inference for single-cell transcriptomics*.* *BMC Genomics*, (2018). 477. doi:10.1186/s12864-018-4772-0

[21] Efremova M, et al., CellPhoneDB: inferring cell-cell communication from combined expression of multi-subunit ligand-receptor complexes*.* *Nat Protoc*, (2020). 1484-1506. doi:10.1038/s41596-020-0292-x
